# Genome-Wide Mutagenesis Reveals That ORF7 Is a Novel VZV Skin-Tropic Factor

**DOI:** 10.1371/journal.ppat.1000971

**Published:** 2010-07-01

**Authors:** Zhen Zhang, Anca Selariu, Charles Warden, Grace Huang, Ying Huang, Oluleke Zaccheus, Tong Cheng, Ningshao Xia, Hua Zhu

**Affiliations:** 1 Department of Microbiology and Molecular Genetics, UMNDJ-Newark, Newark, New Jersey, United States of America; 2 National Institute of Diagnostics and Vaccine Development in Infectious Diseases, Xiamen University, Xiamen, China; University of North Carolina Chapel Hill, United States of America

## Abstract

The Varicella Zoster Virus (VZV) is a ubiquitous human alpha-herpesvirus that is the causative agent of chicken pox and shingles. Although an attenuated VZV vaccine (v-Oka) has been widely used in children in the United States, chicken pox outbreaks are still seen, and the shingles vaccine only reduces the risk of shingles by 50%. Therefore, VZV still remains an important public health concern. Knowledge of VZV replication and pathogenesis remains limited due to its highly cell-associated nature in cultured cells, the difficulty of generating recombinant viruses, and VZV's almost exclusive tropism for human cells and tissues. In order to circumvent these hurdles, we cloned the entire VZV (p-Oka) genome into a bacterial artificial chromosome that included a dual-reporter system (GFP and luciferase reporter genes). We used PCR-based mutagenesis and the homologous recombination system in the *E. coli* to individually delete each of the genome's 70 unique ORFs. The collection of viral mutants obtained was systematically examined both in MeWo cells and in cultured human fetal skin organ samples. We use our genome-wide deletion library to provide novel functional annotations to 51% of the VZV proteome. We found 44 out of 70 VZV ORFs to be essential for viral replication. Among the 26 non-essential ORF deletion mutants, eight have discernable growth defects in MeWo. Interestingly, four ORFs were found to be required for viral replication in skin organ cultures, but not in MeWo cells, suggesting their potential roles as skin tropism factors. One of the genes (ORF7) has never been described as a skin tropic factor. The global profiling of the VZV genome gives further insights into the replication and pathogenesis of this virus, which can lead to improved prevention and therapy of chicken pox and shingles.

## Author Summary

The Varicella Zoster Virus (VZV) is the causative agent of chicken pox and shingles. The long-term efficacy of the current chickenpox vaccine is yet to be determined, and the current shingles vaccine fails to provide protective immunity for a substantial number of individuals. Shingles can also lead to post-herpetic neuralgia (PHN), a debilitating condition associated with an intractable pain that can linger for life. Therefore, VZV remains an important public health concern. We use growth-rate analysis of our genome-wide deletion library to determine the essentiality of all known VZV genes, including novel annotations for 51% of the VZV proteome. We also discovered a novel skin-tropic factor encoded by ORF7. Overall, our identification of genes essential for VZV replication and pathogenesis will serve as the basis for multiple in-depth genetic studies of VZV, which can lead to improved prevention and therapy of chicken pox and shingles. For example, essential genes may be appealing drug targets and genes whose deletion causes a substantial growth defect may be prospective candidates for novel live attenuated vaccines.

## Introduction

Human varicella-zoster virus (VZV) is a widespread human alpha-herpesvirus, and the majority of the US population has been previously exposed [1]. VZV is the causative agent of chicken pox and shingles, the latter of which is associated with a significant incidence of post-herpetic neuralgia [2], [3]. A universal chicken pox vaccine (v-Oka strain) was first introduced to the United States in 1995, and this immunization program has dramatically reduced chicken pox incidence [4]–[10]. However, outbreaks of chicken pox are still seen [11]–[13], and shingles remains an important concern because the current shingles vaccine only reduces the risk of infection by about 50% [14]. Therefore, VZV is still an important pathogen and remains a public health concern in the U.S. [7], [15]. A better understanding of the biology and pathogenesis of VZV is essential to improve the medical prevention and the treatment of VZV infections.

VZV is the smallest member of the human herpesvirus family, with a linear double-stranded DNA genome (125 kb) that encodes 70 unique ORFs. As a result of the recent development of a VZV cosmid system and of the severe combined immunodeficient mouse model with xenografts of human tissue (SCID-hu), many viral ORFs have been investigated in both biochemical and functional studies, shedding light upon several VZV gene functions [16]–[18]. However, the majority of VZV's 70 unique ORFs have not been studied, and their roles in viral replication and cell-/tissue-specific pathogenesis remain unclear. This is partly due to the absence of an efficient genetic tool to quickly isolate a large number of mutants and a true animal model to screen for *in vivo* virulence factors on a large scale [2]. Though the functions of many ORFs can only be predicted based on their homologies to other herpesviruses, such as herpes simplex virus 1, our direct manipulation of VZV's ORFs has enabled us to provide functional annotations for the entire VZV genome.

**Figure 1:**
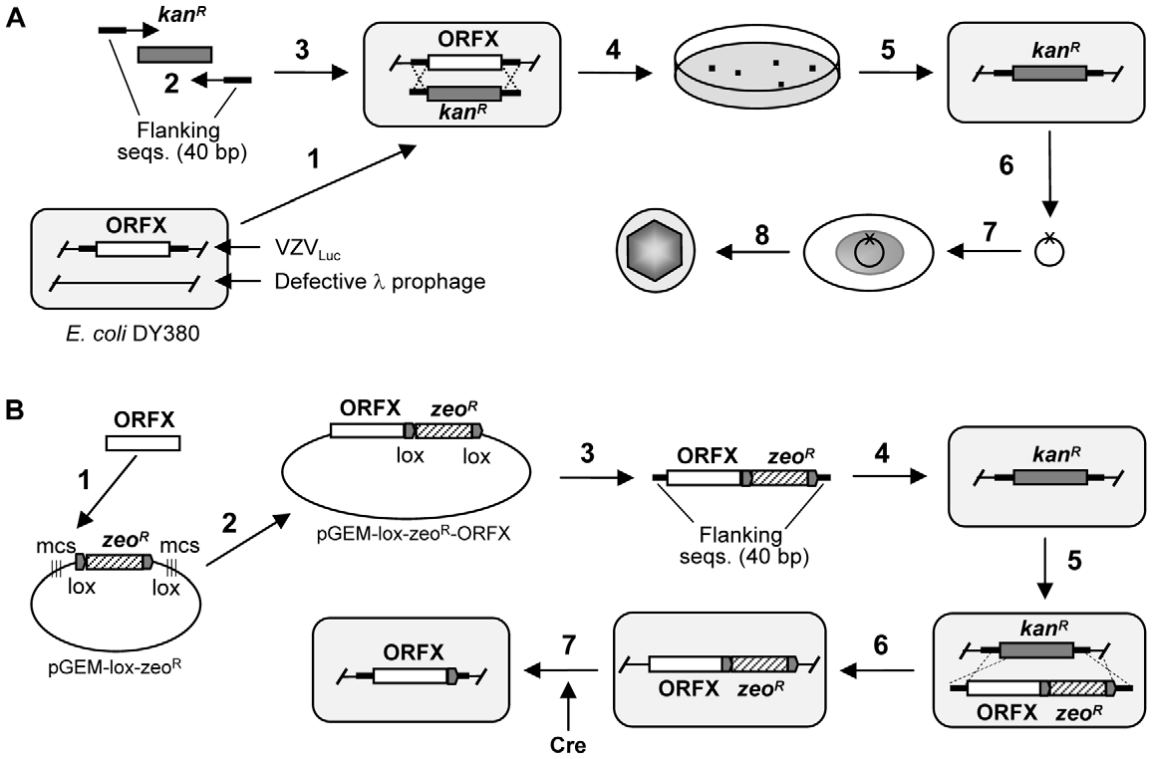
Generation of VZV deletion and rescue clones. A. Generation of the ORFX deletion mutant clone. 1. The *E. coli* DY380 strain provides a highly efficient homologous recombination system, which allows recombination of homologous sequences as short as 40bp. The homologous recombination system is strictly regulated by a temperature-sensitive repressor, which permits transient switching-on by incubation at 42°C for 15min. VZV_luc_ BAC DNA is introduced into DY380 by electroporation. Electro-competent cells are prepared with homologous recombination system activation. 2. Amplification of the kan^R^ expression cassette by PCR using a primer pair adding 40-bp homologies flanking ORFX. 3. About 200ng of above PCR product are transformed into DY380 carrying the VZV_luc_ BAC via electroporation. 4 and 5. Homologous recombination between upstream and downstream homologies of ORFX replaces ORFX with the Kan^R^ cassette, creating the ORFX deletion VZV clone. The recombinants are selected on LB agar plates containing kanamycin at 32°C. 6. The deletion of ORFX DNA is isolated and confirmed by testing antibiotic sensitivity and PCR analysis. The integrity of the viral genome after homologous recombination is examined by restriction enzyme digestion. 7. Purified BAC DNA is transfected into MeWo cells. 8. 3–5 days after transfection the infected cells are visualized by fluorescence microscopy. B. Generation of ORFX rescue virus. 1. To generate the ORFX clone, ORFX was amplified by PCR from the wild-type VZV BAC DNA. 2. ORFX was directionally cloned into plasmid pGEM-lox-zeo to form pGEM-zeo-ORFX. 3. Amplfication of the ORFX-Zeo^R^ cassette by PCR using a primer pair adding 40 bp homologies flanking ORFX. 4. The PCR product was transformed into DY380 carrying the VZV_Luc_ ORFX deletion via electroporation. 5 and 6. Homologous recombination between upstream and downstream homologies of ORFX replaced Kan^R^ with the ORFX-Zeo^R^ cassette, creating the ORFX rescue clone. 7. Zeo^R^ and BAC vector sequences were removed while generating virus from BAC DNA (by co-transfecting a Cre recombinase-expressing plasmid).

The knowledge of VZV replication and pathogenesis is limited, in part because of its highly cell-associated nature in cultured cells and the difficulty of generating recombinant viruses. In order to circumvent some of these problems, we cloned the VZV (p-Oka strain) genome as a bacterial artificial chromosome (BAC) carrying both green fluorescent protein (GFP) and luciferase reporter genes [19]. We then systematically deleted every open reading frame in the VZV genome. An overview of our method for genome-wide mutagenesis is shown in Figure 1. With a highly efficient homologous recombination system and the dual-reporter system, the recombinant viruses were isolated and analyzed.

Human fetal skin organ culture (SOC) has been previously established to mimic VZV skin infection, which allows for the study of VZV replication and pathogenesis [20]. We further combined SOC with the luciferase assay-based viral detection method to facilitate screening of skin tropism determinants. Although many investigators utilize SCID-hu models (grafts of human tissue in severe combined immunodeficient mice) to study VZV pathogenesis *in vivo* [21], SOC is a more suitable and cost efficient approach for genome-wide screening the VZV mutant phenotypes. Nevertheless, any interesting findings can be further verified by further in-depth SCID-hu model studies.

The luciferase VZV BAC (VZV_Luc_) was used to individually delete and/or mutate each of the 70 unique ORFs by employing the *E. coli* DY380 strain recombination system [22]. As a result, a library of whole-ORF deletion mutants was created. Each mutant DNA obtained from *E. coli* was transfected into human melanoma (MeWo) cells, and the results provide direct evidence of that 44 ORFs are essential for viral replication in cultured MeWo cells and 26 are non-essential. Moreover, among the non-essential gene group, 8 ORF deletion mutants showed significant growth defects compared to the wild-type strain (p-value <6.07×10^−21^; see “Statistical Analysis of Mutant Growth Kinetics” section in Materials and Methods), while 18 ORFs were dispensable. All 26 non-essential ORF deletion mutant VZV variants obtained have been tested in SOC. Interestingly, four ORFs were found to be required for optimal viral replication in cultured skin tissue samples, but not in MeWo cells, suggesting their potential roles as skin tropism factors. The results obtained from this study are in agreement with most of those regarding these particular ORFs that have been published to date, and we have provided explanations of all possible discrepancies in the literature. Overall, we provide 51% novel functional annotations to the VZV proteome (36 ORFs).

## Results

### Generation of VZV ORF deletion and rescue mutants

All VZV ORF deletion mutants were constructed from BAC mutants with a luciferase reporter (VZV_Luc_) using a PCR-based approach [19], [22] (also see Supplementary Text S1). Construction of ORF rescued BAC mutants was carried out by adapting a two-step homologous recombination approach in *E. coli* [19], [22] (also see Supplementary Text S1). The generation of a rescue virus is important in order to prove that the deleted fragment was responsible for any growth defect observed in analyses of the mutants. The rescue virus should be able to fully restore the wild-type phenotypes. Because of the large number of ORFs, we chose a small subset of VZV open reading frames to rescure and we have shown these rescue mutants behave as the wild –type strain. A detailed description of these protocols is provided in [19], [22] and an overview is shown in Figure 1. Previous studies in our laboratory have shown that the BAC mutant has an identical growth curve to the wild-type virus [19] and that addition of the luciferase reporter to the BAC virus does not change its growth properties [22].

### Identification of essential VZV genes

**Figure 2:**
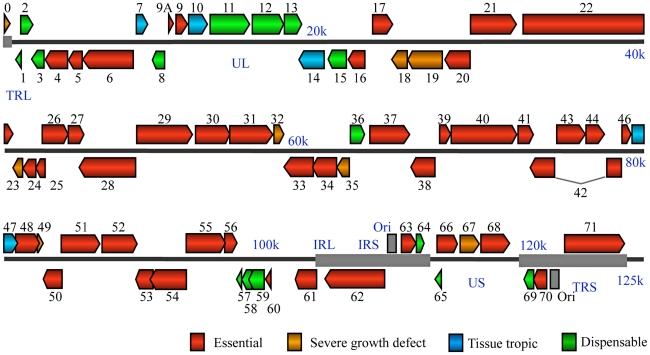
VZV genome-wide functional profiling based on analysis of viral mutants with single open reading frame deletion mutants. Genomic organization and ORFs arrangement are based on the viral sequence of the VZV pOka strain. Each VZV ORF is color-coded according to the growth properties of its corresponding virus gene-deletion mutant in cultured MeWo cells and human fetal skin organ cultures. The grey lines for ORF42 represent a splicing junction. For all growth curves, wild-type infections served as positive controls and mock infections served as negative controls.

All of VZV's 70 unique ORFs were deleted and analyzed based on a bioluminescence detection method, as described previously [19]. For 14 ORFs that overlap with adjacent ORFs (ORF8, ORF9A, ORF25, ORF26, ORF27, ORF28, ORF46, ORF47, ORF48, ORF49, ORF50, ORF54, ORF59 and ORF60), respective partial ORF deletions have been constructed and analyzed. A detailed description of these partial ORFs is included in Supplementary Table S2. The results suggest that 44 ORFs are essential for viral replication in cultured MeWo cells (Table 1 and Figure 2). We have confirmed that ORF4 and ORF5 are essential by making genetic rescue viruses. For the essential group, we provide novel functional annotations for 31 of 44 ORFs. All of these VZV essential genes have HSV-1 homologies (Table 1), and the majority of them are conserved among other herpesviruses. These ORFs encode important viral structural proteins, enzymes involved in DNA replication, and transcriptional regulatory proteins.

**Table 1:** A list of VZV pOka strain ORFs categorized by the growth properties of their respective deletion mutants in MeWo cells and human fetal skin organ cultures (SOC).

ORF	Function	HSV-1 Homology	ORF	Function	HSV-1 Homology
***No growth (44 mutants; Essential in MeWo)***			***No growth (continued)***		
ORF4^R^	Transcriptional regulator	UL54	ORF56^S, 1^	Unknown	UL4
ORF5^R^	Glycoprotein gK^3^	UL53	ORF60^S^	Glycoprotein gL	UL1
ORF6^1^	Primase^3^	UL52	ORF61^2^	Transcriptional regulator	ICP0
ORF9A^S, 2^	Envelope glycoprotein protein gN^3^	UL49A	ORF62/71^D^	Transcriptional regulator	ICP4
ORF9	Tegument pr with unknown function	UL49	ORF63/70^D^	Host range factor; Tegument protein	US1
ORF16^1^	DNA polymerase^3^	UL42	ORF66^2^	Serine-threonine kinase	US3
ORF17^2^	Host shut-off factor	UL41	ORF68	Glycoprotein gE	US8
ORF20^1^	Component of intercapsomeric triplex^3^	UL38	***Growth Defect (8 Mutants; Defect in MeWo and SOC)***		
ORF21	Nucleocapsid protein	UL37	ORF0^R^	Putative transmembrane protein^3^	None
ORF22^1^	Tegument protein with unknown function^3^	UL36	ORF18^1^	Small subunit of ribonucleotide reductase	UL40
ORF24^1^	Phosphoprotein^3^	UL34	ORF19	Large subunit of ribonucleotide reductase	UL39
ORF25^S, 1^	DNA packaging protein	UL33	ORF23	Small capsid protein	UL35
ORF26^S, 1^	DNA packaging protein	UL32	ORF32^1^	Probable substrate for ORF47 kinase^3^	None
ORF27^S, 1^	Nuclear matrix protein^3^	UL31	ORF35	Role in cell to cell fusion	UL24
ORF28^S, 1^	DNA polymerase catalytic subunit	UL30	ORF49^S^	Role in virion envelopment	UL11
ORF29	Single-stranded DNA-binding protein	UL29	ORF67	Glycoprotein gI	US7
ORF30^1^	DNA packaging protein	UL28	***Skin-Tropic (4 mutants; Defect in SOC; WT in MeWo)***		
ORF31^1^	Envelope glycoprotein gB	UL27	ORF7^R, 1^	Tegument protein with unknown function^3^	UL51
ORF33^1^	Major capsid scaffold protein	UL26	ORF10^R^	Tegument protein; transactivator of IE genes	UL48
ORF33.5^1^	Minor capsid scaffold protein	UL26.5	ORF14	Glycoprotein gC	UL44
ORF34^1^	DNA packaging protein	UL25	ORF47^S^	Serine-threonine kinase	UL13
ORF37^1^	Glycoprotein gH	UL22	***Dispensable (14 mutants; WT growth in SOC and MeWo)***		
ORF38^1^	Tegument protein with unknown function^3^	UL21	ORF1	Transmembrane protein	None
ORF39^1^	Integral membrane protein^3^	UL20	ORF2	Unknown	None
ORF40^1^	Component of hexons and pentons	UL19	ORF3	Unknown	UL55
ORF41^1^	Minor capsid protein^3^	UL18	ORF8^S^	Deoxyuridine triphosphatase	UL50
ORF42/45^1^	DNA packaging protein	UL15	ORF11	Tegument protein with unknown function^3^	UL47
ORF43^1^	DNA packaging protein	UL17	ORF12	Tegument protein with unknown function^3^	UL46
ORF44^1^	Tegument pr with unknown function^3^	UL16	ORF13	Thymidylate synthase	None
ORF46^S, 1^	Tegument pr with unknown function^3^	UL14	ORF15^1^	Integral membrane protein^3^	UL43
ORF48^S, 1^	Deoxyribonuclease^3^	UL12	ORF36^1^	Deoxypyrimidine kinase	UL23
ORF50^S, 1^	Glycoprotein gM^3^	UL10	ORF57	Tegument protein; role in virion egress^3^	None
ORF51^1,4^	Origin binding pr/helicase	UL9	ORF58	Unknown	UL3
ORF52^1^	Component of helicase-primase complex^3^	UL8	ORF59^S^	Uracil-DNA glycosylase	UL2
ORF53^1,4^	Tegument protein with unknown function^3^	UL7	ORF64/69^D^	Tegument pr with unknown function^3^	US10
ORF54^S, 1^	DNA packaging protein	UL6	ORF65	Virion protein involved in axonal transport	US9
ORF55,^1^	Helicase^3^	UL5			Essential

Superscript Annotations: “1” corresponds to “results from this study only,” “2” corresponds to “results from this study not consistent with previous studies,” “3” corresponds to “putative function based on homology,” “4” corresponds to “phenotype may be due to effect from adjacent gene,” “S” corresponds to “partial ORF deletion virus study included,” “D” corresponds to “double ORF deletion virus study included,” and “R” corresponds to “Rescue virus study included.”

Among VZV's 44 essential ORFs, the majority encodes proteins with vital functions throughout the viral life cycle. Most VZV proteins that regulate transcription (ORF4, ORF62/71, ORF63/70, and ORF 61) were found to be essential in this study. ORF4 and ORF62/71 are incorporated into the viral tegument, and both encode immediate-early (IE) proteins with transcriptional regulatory activity [23]–[26]. ORF4 and ORF62/71 have been extensively studied, and their essential natures have been suggested previously [27], [28]. Both ORF63/70 and ORF61 encode phosphoproteins primarily localized to the nuclei of infected cells [25]. Although it has been suggested that ORF 63/70 is not essential for viral replication *in vitro* [29], we could not generate a viable virus from a 63/70 double deletion; this result is in agreement with several other studies [30], [31].

Most of the VZV ORFs that encode glycoproteins are essential. Glycoprotein K (gK) (encoded by ORF5) [32], gB (ORF31), gH (ORF37), gM (ORF50) [33], gL (ORF60) [34], [35], and gE (ORF68) [32], [36] are required for viral replication, and many of them had previously been investigated and reported. Only glycoprotein C (ORF14) [37], [38] and gI (ORF67) [36], [39], [40] deletion mutants produced viable viral progenies, and both of these mutants appeared to suffer a severe growth defect. The results regarding the essentiality of VZV glycoprotein genes in this study are in agreement with the published data.

**Figure 3:**
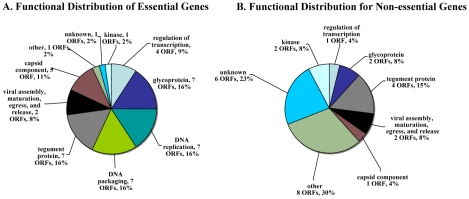
Distribution of functional annotations for essential and non-essential genes. A. Distribution of functional annotations for essential genes. Essential genes are significantly enriched for DNA replication (Bonferroni corrected p-value <10^−4^) and DNA packing (corrected p-value <10^−4^) functional categories. B. Distribution of functional annotations for non-essential genes. Non-essential genes are significantly enriched for other (corrected p-value <10^−3^) and unknown (corrected p-value <0.01) functional categories. Statistical significance was determined by a hypergeometric test.

Essential VZV genes have significantly different enrichment for functional categories than do non-essential genes (Figure 3A). In order to make this calculation, we first listed every gene in a functional category, such as “DNA replication” for a DNA polymerase gene. Then, we compared the proportion of essential (and then of non-essential) genes in each functional category to the background rate expected by chance (e.g. the proportion of genes in that functional category for the entire VZV genome). This calculation was performed using a hypergeometric test. For example, essential genes are significantly enriched for DNA replication (Bonferroni corrected p-value <10^−4^) and for DNA packaging (Bonferroni corrected p-value <10^−4^); ORF28 encodes the catalytic subunit of VZV DNA polymerase and ORF16 encodes the subunit of the viral DNA polymerase processivity factor [2]. DNA binding proteins include proteins encoded by ORF6 (primase), ORF29, ORF33 (capsid protein), ORF41 (capsid protein), ORF51 (helicase), ORF52 (component of helicase/primase complex), and ORF55 (component of helicase/primase complex) [41], [42]. Not surprisingly, almost all of the ORFs that encode DNA packaging proteins—including ORF25, ORF26, ORF30, ORF34, ORF42/45, ORF43, ORF54, and nucleocapsid proteins including ORF21, ORF33.5, and ORF40—also fall into the essential gene category. In contrast, non-essential genes were significantly enriched for other (Bonferroni corrected p-value <10^−3^) and unknown (Bonferroni corrected p-value <0.01) functional categories (Figure 3B).

### Identification of non-essential genes

**Figure 4:**
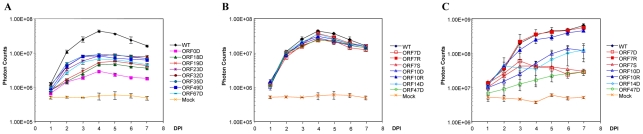
Growth curve analysis of some VZV deletion mutants. A. Eight VZV ORF deletion mutants showing slow growth kinetics in cultured MeWo cells. One hundred PFU of each deletion mutant and VZV_Luc_ (WT) were infected with MeWo cells in 6-well dishes in triplicate. Bioluminescence was measured using the IVIS system every day for 7 days after D-luciferin was applied to the cultured media. Total Photon Count in each well (photons/sec/cm^2^/steradian) was measured, and the values from the triplicate results were averaged. The growth curves were generated when averaged photon counts for each day were plotted. Error bars represent standard deviation for three replicates. B. *In vitro* growth curve analysis of VZV ORF7, ORF10, and ORF47 mutant viruses. One hundred PFU of ORF7 and ORF10 mutant and rescue (ORF7D, ORF7S, ORF7R, ORF10D, and ORF10R) and ORF47 deletion virus (ORF47D) from infected MeWo cells were used to infect 50% confluent MeWos seeded in 6-well dishes. Experiments were performed in triplicates. The growth curves were generated as described in A. C. Growth curve analysis of VZV ORF7, ORF10, and ORF47 mutants in human fetal skin organ cultures. Skin tissues were inoculated with 5×10^3^ PFU VZV_Luc_ or other VZV variants, as indicated. VZV replication was monitored daily by IVIS for one week as bioluminescence emitting from each skin culture was measured. Each line represents an average of the data from 3 different skin tissue samples, all infected with the same virus. D-luciferin was also applied to three uninfected skin tissue samples (injected with PBS) as mock infection.

In this study, we found that 26 ORFs are non-essential genes and 6 of these lack HSV-1 homologies (ORF0, ORF1, ORF2, ORF13, ORF32, and ORF57) (Table 1). According to the growth kinetics (in cultured MeWo cells), 8 ORF mutants had significant growth defects (p-value <6.07×10^−21^), and the peak signals of the viral detection assay were at least 5-fold less than were those of the wild-type parental strain (Figure 4A). Two of these VZV ORF deletion growth phenotypes, ORF18 and ORF32, have not been previously reported, and two others (ORF23 and ORF35 deletions) have been confirmed to have growth defects *in vitro*, which is in accordance with previously published data [43], [44]. ORF0 deletion's growth defect has been confirmed by making its genetic revertant [19]. ORF18 and ORF19 respectively encode the small and large subunits of ribonucleotide reductase, and both of them diminished viral growth when deleted in this study. The result on ORF19 is in accordance with previous publications [45]. ORF32 encodes a phosphoprotein that is post-translationally modified by ORF47 protein kinase [46]. Among these 8 viral mutants showing severe growth defects, atypical morphology of virally infected cells was frequently observed, including reduced plaque sizes and altered syncytia formation.

The remaining 18 VZV ORFs had wild type growth curves for viral replication in cultured MeWo cells. *In vitro* growth curve analysis showed that these ORF deletion mutants had the same growth kinetics as their wild-type parental strain, VZV_Luc_ (Table 1). Previous studies have reported that 15 of these genes (ORF1, ORF2, ORF3, ORF8, ORF10, ORF11, ORF12, ORF13, ORF14, ORF47, ORF57, ORF59, ORF58, ORF64/69, and ORF65) are non-essential [17], [31], [38], [46]–[54]. In this study, three of these ORF mutants (ORF7, ORF15, and ORF36) have been shown to be dispensable for *in vitro* viral replication for the first time.

The human fetal skin organ culture (SOC) model has been proven to be a simple and convenient alternative to the SCID-hu mouse model in the study of VZV pathogenesis [20], especially in the case of an initial genome-wide screening for skin tropism determinants. Although 26 VZV ORFs were found to be non-essential for viral replication in cultured MeWo cells, it was possible that some of these viral genes encode proteins critical for optimal viral infection in skin tissue. To test this hypothesis, all 26 non-essential ORF deletion viruses were further analyzed in cultured skin-tissue samples.

Every deletion mutant that showed severe growth defects in cultured MeWo cells also demonstrated significantly slow growth kinetics in human skin samples when compared to wild-type VZV (p-value <9.05×10^−19^). Only two of these genes (ORF35 and ORF67) have been previously reported to be required for viral growth in SCID-hu skin mouse models [40], [44]. Therefore, we have been able to provide novel functional annotations for the other 6 deletion mutants with severe growth defects.

Many non-essential genes appear to cluster together, particularly between ORF0 to ORF15 (Figure 2). More than 70% of ORFs (12 out of 17) in this region are non-essential, compared to 37% of the entire genome. Four out of six VZV ORFs without HSV-1 homologues are also located in this region, so this region may be more evolutionarily variable compared to other highly conserved regions

Among the 18 VZV ORFs dispensable for viral replication in cultured MeWo cells, 14 were also dispensable for viral replication in skin tissue (Table 1). Among the above 14 deletion mutants, we have been able to provide novel *ex vivo* functional annotations for all but one of these 18 genes (ORF64/69) [31].

### Identification of skin-tropic genes

Interestingly, among non-essential VZV ORFs, four ORFs (ORF7, ORF10, ORF14, and ORF47) appear to have selective impacts on viral replication in skin tissue. The growth of each virus in SOC was compared with its growth in cultured MeWo cells. These ORF deletion mutants grew like the wild-type strain *in vitro* (Figure 4B). In contrast, they showed significant growth defects in skin organ cultures (p-value <9.51×10^−19^; Figure 4C). For instance, the ORF10 deletion mutant had a growth defect in SOC. The bioluminescence signal kept increasing during the entire 7-day experiment period; the total photon count values consistently remained approximately 10-fold less than those of the wild-type strain. The ORF7 deletion mutant virus quickly reached its growth peak around 3 days after inoculation, and then bioluminescence steadily declined. To prove that the VZV ORF7 and ORF10 growth defects observed in cultured skin-tissue samples were due to the functions of the deleted genes rather than to undesirable mutations in other regions of the genome, rescue viruses ORF7R and ORF10R were generated. The growth curve analysis indicated that ORF7R and ORF10R viruses grew in MeWo cells indistinguishably from wild-type VZV, as expected (Figure 4B). In skin organ cultures, they were also able to fully recover the growth defects of the corresponding deletion mutant viruses and grew as well as the wild-type strain (Figure 4C). In contrast, ORF47 deletion virus had a more severe growth defect, approximately 80–100 fold (2 log) less than wild-type VZV. Our results suggest these three ORFs are important for viral replication in human skin tissue but not in cultured MeWo cells.

Three skin-tropic virulence factors (ORF10, ORF14, and ORF47) have been previously identified. VZV ORF10 encodes a tegument protein that enhances transactivation of VZV genes, and it was shown to be dispensable for VZV replication *in vitro* [47]. Recent studies showed that ORF10 protein is required for efficient VZV virion assembly and is a specific determinant of VZV virulence in SCID-hu skin xenografts but not in human T cells *in vivo* [55], [56]. ORF14 (gC) has been reported to have reduced infectivity in an SCID-hu skin model [38]. VZV ORF47 encodes a serine/threonine protein kinase and was shown to be dispensable for viral replication in cultured MeWo cells [48]. It has been designated as a virulence factor for both skin tissue and T cells in SCID-hu models [38]. The findings of these three skin-tropic ORFs not only confirmed previous studies but also further verified the similarity between SCID-skin and SOC systems.

In the current study, ORF7 has been identified as a novel skin-tropic virulence factor. In order to confirm the accuracy of our results, we also produced a premature stop-codon mutant (ORF7S) by mutating the 5^th^ codon from TGT to the TGA stop codon (see Table S1). Just like ORF7D, ORF7S showed wild-type growth in MeWo (Figure 4B) but had a growth defect in SOC (Figure 4C).

## Discussion

In this study, a global functional analysis of the entire VZV genome was performed that emphasized the identification of viral ORFs important for viral replication both in cultured MeWo cells and human fetal skin organs. We took full advantage of the highly efficient luciferase VZV BAC system and obtained a library of single ORF deletion mutants. Advanced live culture bioluminescence imaging technology allowed us to systematically test a large number of mutant viruses for comparing viral growth kinetics in different systems.

VZV has a 125-kb DNA genome encoding 70 unique open reading frames (Table 1, Figure 2). In this study, all of the predicted 70 ORFs were individually deleted. Our results directly showed that 44 ORFs encode essential genes and 26 ORFs encode non-essential genes. Among the non-essential group, 8 ORF deletion mutants suffered severe growth defects in MeWo cells. Fourteen ORFs were shown to be dispensable for viral replication, both in MeWo cells and in SOC. We also found 4 tissue tropic factors (ORF7, ORF10, ORF14, and ORF47) that showed a growth defect in SOC but normal growth in MeWo. Three of these tissue-tropic factors (ORF10, ORF14, and ORF47) have been previously identified, but ORF7 has never been previously studied.

In the current study, we have reported ORF7 as a novel VZV skin-tropic factor. ORF7 encodes a 29-kDa tegument protein, and its function remains unknown. The homolog of the VZV ORF7 protein in the herpes simplex virus is the UL51 protein. Recent studies showed that deletion of HSV-1 UL51 causes reduced size plaque formation and low infectivity [56]. Similarly, the function of the UL51 gene product of the pseudorabies virus (PrV) has been investigated by generating a deletion mutant, and the result suggested that the UL51 protein is involved in viral egress, but is not essential for viral replication [57]. Our result suggests that VZV ORF7 might serve as a skin-specific virulence factor. However, the role of ORF7 in pathogenesis needs further investigation.

Despite the large differences between herpesvirus genomes (ranging from 125 kb to >230 kb), all the herpes viruses studied thus far have a similar number of essential genes. For example, HSV-1 encodes 37 essential genes and 48 non-essential genes [58]; human cytomegalovirus (HCMV), which is one of the largest human DNA viruses, encodes 45 essential genes and 117 non-essential genes [59]. Our data suggest that VZV, which contains the smallest genome, encodes 44 essential genes and 26 non-essential genes. A comparison between the essentiality of HSV-1 and HCMV homologues to essential VZV genes is provided in Supplementary Table S3. Of the 44 essential genes, 26 have essential homologues in HSV, and all essential gene homologues conserved in CMV (18 of 44 essential VZV genes) are essential. Therefore, we believe that several of these essential genes perform core functions for all of these herpesviruses.

Unlike the other functional profiling studies performed on HCMV [59], our results did not reveal any VZV-encoded factors that repress viral replication in cultured MeWo cells or in human fetal skin tissue. If such VZV temperance genes existed, enhanced growth kinetics should have been observed by making the corresponding ORF deletion mutants.

There is also an apparent size difference between essential and non-essential ORFs. Essential ORFs are significantly larger in size compared to non-essential ones (μ = 1250 bp vs. μ = 970 bp, respectively, p = 6×10^−4^ by t-test). The 10 largest VZV ORFs are all essential, while 8 out of 11 VZV ORFs less than 600 bp are non-essential.

All of our results are in agreement with previous VZV functional annotations, except for those on ORFs 9A, 17, 61 and 66, for which we could not generate viral deletion mutant progenies with sufficient titers for growth studies. For example, previous studies indicated that was ORF9A *not* essential viral growth in cell culture (due to insertion of a premature stop codon) *yet* they also showed that failure to express either of these genes resulted in growth defects [60]. Therefore, we believe that our findings are at least in partial agreement with previous studies because this previous study utilized a premature stop codon (thus allowing expression of a partial protein), whereas we completely removed ORF9A from the VZV genome.

Although some studies have shown ORF17 to be dispensable for viral replication [61], other studies have shown the gene to be essential for growth under certain conditions [62]. Therefore, we believe this discrepancy can probably be explained by subtle differences in experimental design (such as the temperature of the growth culture, as described in [62], and we believe that our analysis for ORF17 deletion best reflects conditions *in vivo*.

ORF61 has also been suggested to be a non-essential gene for viral replication *in vitro* in a previous study [63], [64]. However, we could not retrieve enough infectious viral progeny from the ORF61 deletion clone, even after repeated transfection and extensive incubation. Large deletion mutants of ORF61 [63] and promoter bashing experiments [64] have shown ORF61 to be important for viral replication (albeit non-essential) due to a considerable growth defect shown in the deletion. However, no complete deletion virus has ever been created, so it is possible that the large deletions may have only been sufficient to cause a growth defect, whereas our complete deletion results in a complete loss of VZV replication.

ORF66 has been previously cited as dispensable for viral replication, but we have found it to be essential [65]–[67]. In previous studies, a premature stop codon mutant of ORF66 resulted in a decrease in viral titer, but not in a complete loss of viral replication [65], [66]. Premature stop codons were inserted such that more than 50% of the original coding sequence remained and was able to be expressed, so we believe this discrepancy can be explained by the possible attenuated activity of the partial protein (which did have a substantial growth defect), while our ORF66 deletion removed the entire sequence. For the cosmid-based studies [67], [68], a premature stop codon mutant (with a 21-amino acid partial protein expressed) had to be used to assess the impact of ORF66 on viral replication. However, the authors [68] were also unable to produce infectious virus with a complete ORF66 deletion mutant (which is identical to our results).

In this study, we have presented novel functional annotations for 36 VZV genes. Due to the global nature of our study and the lack of well-defined upstream and downstream regulatory regions for most VZV genes, some of our annotations may have to be redefined by more detailed studies (genes most likely to be affected by adjacent genes are specifically noted in Table 1). Moreover, the current profiling study has provided the first global view of VZV genomic functions in viral replication, which is likely to serve as the basis for further investigative studies on many VZV genes.

## Materials and Methods

### Cells, virus and PCR primers

Human melanoma (MeWo) cells were grown in DMEM supplemented with 10% fetal calf serum, 100U of penicillin-streptomycin/ml, and 2.5ug of amphotericin B/ml, as previously described, and used to propagate VZV *in vitro* [18], [69]. VZV_Luc_ containing the entire p-Oka VZV genome was constructed as previously described [19]. Recombinant VZV_Luc_ virus was derived by transfection methods [19], [22] (also see Supplementary Text S1). All primer sequences are listed in Supplementary Table S1. Primer sequences were designed based upon the Dumas VZV strain (Accession Number: NC_001348).

### Growth analysis of viral mutants in vitro

VZV_Luc_ DNAs were transfected into MeWo cells using the FuGene 6 transfection kit (Roche, Indianapolis, IN) [19], [22] (also see Supplementary Text S1). Recombinant viruses were titrated by infectious focus assay. MeWo cells were seeded in 6-well tissue culture plates and inoculated with serial dilutions of VZV-infected MeWo cell suspensions. Plaques were counted by fluorescence microscopy at 3 days after inoculation. All transfections were performed a minimum of 3 times. Since VZV is highly cell-associated under tissue culture conditions, mutant VZV-infected MeWo cells were harvested, titrated and stored in liquid nitrogen. Wild-type infections served as positive controls and mock infections served as negative controls.

*In vitro* growth curve analyses were carried out by live-cell bioluminescence detection assay. MeWo cells were infected with 100 PFU of infected MeWo cell suspensions on 6-well tissue culture plates. Every 24 h, the cell culture medium was replaced with medium containing 150 ug/ml D-luciferin (Xenogen, Alameda, CA). After incubation at 37°C for 10 min, the bioluminescent signals were quantified and recorded using an IVIS Imaging System (Xenogen), following the manufacturer's instructions. After each measurement, the luciferin-containing medium was replaced with fresh cell culture medium. Measurements were taken daily from the same plate for 7 days. Bioluminescence signal data from each sample were quantified by manually demarcating regions of interest and analyzed using LivingImage analysis software (Xenogen). It has been demonstrated previously that both the infectious center assay and the luciferase assay correlate well [19], [22].

### Growth analysis of viral mutants in SOC

Human fetal skin-tissue samples (∼20 weeks gestational age) were acquired from Advance Biosciences Resources (Alameda, CA). Skin organ-culture techniques were as previously described [20]. *Ex vivo* growth curve analyses were carried out by live-tissue bioluminescence assay. Infected MeWo cells were titrated and then re-suspended in skin organ culture media (SOCM). After 24 h of incubation, each skin-tissue section was injected five times with 10 ul of the virus-infected cell suspension (total inoculation was 5×10^3^ PFU per tissue) by a 1-ml syringe fitted with a 27-gauge needle attached to a volumetric stepper (Tridak, Brookfield, CT). After inoculation, the sections were placed individually on 500 um mesh NetWell inserts (Corning, Corning, NY) that rested above 1ml of SOCM in each well of 12-well plates and followed by a 24 h incubation in a tissue culture incubator, 37°C, 5%CO_2_. Each 24 h, SOCM was replaced with media containing 150ug/ml of D-luciferin. Following 10 min incubation at 37°C, the bioluminescence being emitted from individual cultured skin-tissue samples was recorded using the IVIS Imaging System. After the measurements, each sample (still on a NetWell insert) was transferred onto new 12-well plates with fresh SOCM. The measuring process was repeated every 24 h for 7 days. Bioluminescence signals from manually defined regions of interest were quantified and analyzed. All experiments were performed in triplicate. Wild-type infections served as positive controls and mock infections served as negative controls.

### Statistical analysis of mutant growth kinetics

Wild type and mutant growth curves (7 time points, 3 replicates each) were compared using the “timecourse” Bioconductor package [70], [71]. The difference in growth rate for wild type and mutant growth curves was estimated by the mb.long function was used to estimate a Hotelling T^2^ test statistic using the mb.long function. P-values for the T^2^ test statistic were calculated using an F-distribution. The T^2^ test statistic did an excellent job of quantifying the difference in growth curves, but a very strict p-value cutoff was required in order to define statistically significant growth defects (implying that the test statistic may be too sensitive). Therefore, we used a Mann-Whitney U test in order to determine which individual time points significantly differed between wild type and deletion mutant strains. All strains with reported growth defects have at least 6 significantly reduced time points (p<0.05).

## Supporting Information

Table S1 Sequences of all primers used in VZV genomic functional profiling.
(0.08 MB PDF)

Table S2 Description of Overlapping VZV ORFs.
(0.05 MB PDF)

Table S3 Essentiality of Essential VZV Homologous in HSV and CMV.
(0.05 MB PDF)

Text S1 Supplementary Materials.
(0.03 MB DOC)
