# Supplementary material for: Genome-Wide Mutagenesis Reveals That ORF7 Is a Novel VZV Skin-Tropic Factor
Source: PLoS Pathog. 2010 Jul 1;6(7):e1000971. doi: 10.1371/journal.ppat.1000971 (PMC2895648; doi:10.1371/journal.ppat.1000971)
Supplement: Table S1 — Sequences of all primers used in VZV genomic functional profiling. (0.08 MB PDF) [file ppat.1000971.s001.pdf]

Table S1. Sequences of all primers used in VZV genomic functional profiling

Primer Name	Primer Sequence (5`-3`)	Use
ORF0KanF	AACCCGCGCCTTTTGCGTCCACCCCTCGTTTACTGCTCGGGCTCTTGTTGGCTAGTGCGTA	Amplify KanR with ORF0 flanking homologies
ORF0KanR	GCAAGCGAGAATAAATACCTTCCCCTTCCGGACAGTAGTTTCTGCCAGTGTTACAACCAA	Amplify KanR with ORF0 flanking homologies
ORF1KanF	ACTCAACTACATGAAACTACTGTCCGGAAGGGGAAGGTATGCTCTTGTTGGCTAGTGCGTA	Amplify KanR with ORF1 flanking homologies
ORF1KanR	ATAAGCAAATCCTGTTAATATTATATTTTTGGGATCCGCATCTGCCAGTGTTACAACCAA	Amplify KanR with ORF1 flanking homologies
ORF2KanF	TAATAGCTATTATCGTAACCCACCCCCGTAAAATCATAAAGCTCTTGTTGGCTAGTGCGTA	Amplify KanR with ORF2 flanking homologies
ORF2KanR	AAATACGTACAATCGAAAAAAGGTGTATTTTATTTAGTGATCTGCCAGTGTTACAACCAA	Amplify KanR with ORF2 flanking homologies
ORF3KanF	CTTTTTTCGATTGTACGTATTTTTATTTAAATGTGTAGTTGCTCTTGTTGGCTAGTGCGTA	Amplify KanR with ORF3 flanking homologies
ORF3KanR	GGGTAAAACACACACCAGACGTGTACCGAACGTTTAATTATCTGCCAGTGTTACAACCAA	Amplify KanR with ORF3 flanking homologies
ORF4KanF	TTAGTATGTTTTGACAAGCATGAAAAAGGTATTTTTTATTGCTCTTGTTGGCTAGTGCGTA	Amplify KanR with ORF4 flanking homologies
ORF4KanR	AGGCAACTGCAAACACGCAATTGTCAGATATTTTGCAGCCTCTGCCAGTGTTACAACCAA	Amplify KanR with ORF4 flanking homologies
ORF5KanF	GGCTCACCCAACCCCGCAATGGGCGTGTTTAGTCACATGAGCTCTTGTTGGCTAGTGCGTA	Amplify KanR with ORF5 flanking homologies
ORF5KanR	TGATACTACATCGTGCTTGAATTGCCATCTTCCACGGGTCTCTGCCAGTGTTACAACCAA	Amplify KanR with ORF5 flanking homologies
ORF6KanF	GATGGCAATTCAAGCACGATGTAGTATCACACGGTTGGTGGCTCTTGTTGGCTAGTGCGTA	Amplify KanR with ORF6 flanking homologies
ORF6KanR	ACCGTCTGCATGATTGACTGGCTTTCCAACGTATTGAACTTCTGCCAGTGTTACAACCAA	Amplify KanR with ORF6 flanking homologies
ORF7KanF	GATTTATCCATAGTTCAATACGTTGGAAAGCCAGTCAATCGCTCTTGTTGGCTAGTGCGTA	Amplify KanR with ORF7 flanking homologies
ORF7KanR	AAACATACACCAGAAACGTTTTTAGTTTTTATTTCAATATTCTGCCAGTGTTACAACCAA	Amplify KanR with ORF7 flanking homologies
ORF8/9AKanF	TATAAAATTAAAACATTGCTGGCTGGCGTGGTTATTACATGCTCTTGTTGGCTAGTGCGTA	Amplify KanR with ORF8/9A flanking homologies
ORF8/9AKanR	CCCTCTTATACACGCCTGCCCCTTTTATAGGCAAACGGGTTCTGCCAGTGTTACAACCAA	Amplify KanR with ORF8/9A flanking homologies
ORF9KanF	CGTGTTTGGATATTTCACGACCCTATCGTTTATTTACGTAGCTCTTGTTGGCTAGTGCGTA	Amplify KanR with ORF9 flanking homologies
ORF9KanR	TACATAATACCGGGTAAACCGTTACTGCGTAATTATATCCTCTGCCAGTGTTACAACCAA	Amplify KanR with ORF9 flanking homologies
ORF10KanF	GGGAATCGCTTATTTAAACTAAAGATTTTACTCTATAAGTGCTCTTGTTGGCTAGTGCGTA	Amplify KanR with ORF10 flanking homologies
ORF10KanR	CGTTTTCGTAATTTATTTACACCCTCTACCCCAATGACGTTCTGCCAGTGTTACAACCAA	Amplify KanR with ORF10 flanking homologies
ORF11KanF	GGATGTTTTACAGGCGCGTTTGTTTGTCTCGGTTATAAGTGCTCTTGTTGGCTAGTGCGTA	Amplify KanR with ORF11 flanking homologies
ORF11KanR	GGAAACGTTCTTTTCATCCTAATGAAAAAAATCACAACCCTCTGCCAGTGTTACAACCAA	Amplify KanR with ORF11 flanking homologies
ORF12KanF	GAGGATCGACCTTTACAAGATATAATTTGTCCATATCGCAGCTCTTGTTGGCTAGTGCGTA	Amplify KanR with ORF12 flanking homologies
ORF12KanR	CCAAAGTGCCTAGTCCATAACTATCTATTAACCGCTGTACTCTGCCAGTGTTACAACCAA	Amplify KanR with ORF12 flanking homologies
ORF13KanF	ATCAAGTGGTCGTTTGTATTTAACGATTATTACCGGTACCGCTCTTGTTGGCTAGTGCGTA	Amplify KanR with ORF13 flanking homologies
ORF13KanR	TCGCAACACATCTACTGTCTTGACAACATTTAAAAATCCATCTGCCAGTGTTACAACCAA	Amplify KanR with ORF13 flanking homologies
ORF14KanF	AATAAAATGATATACACAGACGCGTTTGGTTGGTTTCTGTGCTCTTGTTGGCTAGTGCGTA	Amplify KanR with ORF14 flanking homologies
ORF14KanR	TTTATTTAAGGGGAGCGTGGATGTGTCAATAAAAACCAGGTCTGCCAGTGTTACAACCAA	Amplify KanR with ORF14 flanking homologies
ORF15KanF	AAAACGGCTTTTGCAACTTTAAAAACTTTATTTATCTCGAGCTCTTGTTGGCTAGTGCGTA	Amplify KanR with ORF15 flanking homologies
ORF15KanR	GAACACATTTTTATCTGTTTTGAATTATTAAACTTAAGACTCTGCCAGTGTTACAACCAA	Amplify KanR with ORF15 flanking homologies
ORF16KanF	ACACACAAGTTTGTATGTAAAATATAAGCAAAAGTTGCACGCTCTTGTTGGCTAGTGCGTA	Amplify KanR with ORF16 flanking homologies
ORF16KanR	TGATTACTTTTTCTATAGTAAACATTTTAAGTAGTAAATTTCTGCCAGTGTTACAACCAA	Amplify KanR with ORF16 flanking homologies
ORF17KanF	AACCTTTCACAAGACGATATAAACTTGGTTAAGTGTATTGGCTCTTGTTGGCTAGTGCGTA	Amplify KanR with ORF17 flanking homologies
ORF17KanR	AAATAAAACAATGAACCATTAAGTCGCTCTTATGTGTGTTTCTGCCAGTGTTACAACCAA	Amplify KanR with ORF17 flanking homologies
ORF18KanF	ACTTAATGGTTCATTGTTTTATTTTGCTCGTATATACATGGCTCTTGTTGGCTAGTGCGTA	Amplify KanR with ORF18 flanking homologies
ORF18KanR	CAGTTGTGCTTTATAAATTTACACGGGAAACTATTCCAAATCTGCCAGTGTTACAACCAA	Amplify KanR with ORF18 flanking homologies
ORF19KanF	AATCTTTCTGATCCATTTTGGAATAGTTTCCCGTGTAAATGCTCTTGTTGGCTAGTGCGTA	Amplify KanR with ORF19 flanking homologies
ORF19KanR	GTTTAAAGGTTTATTCCGGAGCCTAAATACGTTATCCGTTTCTGCCAGTGTTACAACCAA	Amplify KanR with ORF19 flanking homologies
ORF20KanF	CCCACGCTATGTTAAATTCAGTTTATTTTACATACATGCTGCTCTTGTTGGCTAGTGCGTA	Amplify KanR with ORF20 flanking homologies
ORF20KanR	CCACGTCACGATTGCATTGTGTGAATTTAACCCCTCAGCTTCTGCCAGTGTTACAACCAA	Amplify KanR with ORF20 flanking homologies
ORF21KanF	TCATATCACGTTATAAAGTTAAGTCAGCGTAGAATATACCGCTCTTGTTGGCTAGTGCGTA	Amplify KanR with ORF21 flanking homologies
ORF21KanR	CCACAAATACTTTAATGTACATAAACACGCCTGTTTTATATCTGCCAGTGTTACAACCAA	Amplify KanR with ORF21 flanking homologies
ORF22KanF	GTCCCTACCAGTTGAGCGCGTAATTTTCGTAAGCAATAAAGCTCTTGTTGGCTAGTGCGTA	Amplify KanR with ORF22 flanking homologies
ORF22KanR	GAAAATAATCGTTGTTTTTTTTTCTTTAATAGGCGCTACTTCTGCCAGTGTTACAACCAA	Amplify KanR with ORF22 flanking homologies
ORF23KanF	AAAGAAAAAAAAACAACGATTATTTTCTGTGTATTTTTATGCTCTTGTTGGCTAGTGCGTA	Amplify KanR with ORF23 flanking homologies
ORF23KanR	CGTGTATGCTGTTATCGATTGTCCCGTAAACTAATAAACGTCTGCCAGTGTTACAACCAA	Amplify KanR with ORF23 flanking homologies

Table S1. Sequences of all primers used in VZV genomic functional profiling (cont.)

Primer Name	Primer Sequence (5`-3`)	Use
ORF24KanF	ATACACGTACATCTGCGCAGGATATGTACGGAAAGGCAATGCTCTTGTTGGCTAGTGCGTA	Amplify KanR with ORF24 flanking homologies
ORF24KanR	TACTTCACGCGTGTGCCTTGGTTTGCAACCTTAGATCAATTCTGCCAGTGTTACAACCAA	Amplify KanR with ORF24 flanking homologies
ORF25KanF	CCCCCTGACGACTTATTAATGCGTTTATTTTCCCCATGTAGCTCTTGTTGGCTAGTGCGTA	Amplify KanR with ORF25 flanking homologies
ORF25KanR	TAACAAATCGTTTTTTAGCAGAAGGGCTGGATTATAAGCGTCTGCCAGTGTTACAACCAA	Amplify KanR with ORF25 flanking homologies
ORF26KanF	ATCGGTGTCTGTAATTAAATCGCGGGCCCTCATCCCAGAGGCTCTTGTTGGCTAGTGCGTA	Amplify KanR with ORF26 flanking homologies
ORF26KanR	ACGCATCGAAGGCCCGCTCCGAGATACGGATTTATATCGCTCTGCCAGTGTTACAACCAA	Amplify KanR with ORF26 flanking homologies
ORF27KanF	TTTGAAGGTCGTGTATGTGCTGGCCTGTGGACATTGGCGTGCTCTTGTTGGCTAGTGCGTA	Amplify KanR with ORF27 flanking homologies
ORF27KanR	GCCCCGCGCTGGGATAATAAAATGAACACTGAAGCTGAAATCTGCCAGTGTTACAACCAA	Amplify KanR with ORF27 flanking homologies
ORF28KanF	TCCATTGGAACATCGGACGTTTACTGTAAAATGTGTGACCGCTCTTGTTGGCTAGTGCGTA	Amplify KanR with ORF28 flanking homologies
ORF28KanR	AACACTGGCTTTTTACAAGATTATCAATCGTTAACATAAATCTGCCAGTGTTACAACCAA	Amplify KanR with ORF28 flanking homologies
ORF29KanF	CCTGCCAACAACCCCCCATTATTACGAGTACTTCACCAAAGCTCTTGTTGGCTAGTGCGTA	Amplify KanR with ORF29 flanking homologies
ORF29KanR	ACTCACCCGCGTGTGGGCTTTAATTGGATAAAGAGGGAGGTCTGCCAGTGTTACAACCAA	Amplify KanR with ORF29 flanking homologies
ORF30KanF	ATTCAGCTTACAGTCCAGTGGACAGTAACAGCCCGATAACGCTCTTGTTGGCTAGTGCGTA	Amplify KanR with ORF30 flanking homologies
ORF30KanR	AATATGCATAAAAAGCATAACCACACCCCCGTAACGGAGGTCTGCCAGTGTTACAACCAA	Amplify KanR with ORF30 flanking homologies
ORF31KanF	CGTTACGGGGGTGTGGTTATGCTTTTTATGCATATTTTCTGCTCTTGTTGGCTAGTGCGTA	Amplify KanR with ORF31 flanking homologies
ORF31KanR	CACATTTTTATTAATAAATTAAAACAAACCCCCTGGCTATTCTGCCAGTGTTACAACCAA	Amplify KanR with ORF31 flanking homologies
ORF32KanF	TTGTACCTCTACCGCACCCGATATCTTAACGGGGGTTATTGCTCTTGTTGGCTAGTGCGTA	Amplify KanR with ORF32 flanking homologies
ORF32KanR	AGGCGTTTTTATGGGTGGTAAGGTTTTATTTAAGTTAAATTCTGCCAGTGTTACAACCAA	Amplify KanR with ORF32 flanking homologies
ORF33.5/33KanF	CAAACACGCCTTTTATGAATGTAATACTTTTATTTGTTGGGCTCTTGTTGGCTAGTGCGTA	Amplify KanR with ORF33.5/33 flanking homologies
ORF33.5/33KanR	CACCGTGAGTATTCACTATCCGGTCCGTGGGGTGTTTATATCTGCCAGTGTTACAACCAA	Amplify KanR with ORF33.5/33 flanking homologies
ORF34KanF	GCAGCCATTATAAACACCCCACGGACCGGATAGTGAATACGCTCTTGTTGGCTAGTGCGTA	Amplify KanR with ORF34 flanking homologies
ORF34KanR	TTTATATTCACGCCAGTTGACTTTGTTTGCTGCAGACACCTCTGCCAGTGTTACAACCAA	Amplify KanR with ORF34 flanking homologies
ORF35KanF	GGCGTGAATATAAACCGGTACTGCTTTAAAAGCTGTTTTCGCTCTTGTTGGCTAGTGCGTA	Amplify KanR with ORF35 flanking homologies
ORF35KanR	AAACGCGTCTTAGGTTATCTTCTGGGACGGAACTTCAAATTCTGCCAGTGTTACAACCAA	Amplify KanR with ORF35 flanking homologies
ORF36KanF	TCCCAGAAGATAACCTAAGACGCGTTTGTCTACAATAAACGCTCTTGTTGGCTAGTGCGTA	Amplify KanR with ORF36 flanking homologies
ORF36KanR	ATCATCTTTTTACTGGTACATACGTAAATACTAGGTATATTCTGCCAGTGTTACAACCAA	Amplify KanR with ORF36 flanking homologies
ORF37KanF	ATAACGTTGCGGTGATATTGTAGCGCAAGTAACAGCGACTGCTCTTGTTGGCTAGTGCGTA	Amplify KanR with ORF37 flanking homologies
ORF37KanR	AGAGAATACGTTTATAGTGACTTTTTATTATACATGTTTTTCTGCCAGTGTTACAACCAA	Amplify KanR with ORF37 flanking homologies
ORF38KanF	AACGTATTCTCTACAATACTTTATTCGCGAATAATACACAGCTCTTGTTGGCTAGTGCGTA	Amplify KanR with ORF38 flanking homologies
ORF38KanR	ACGGCAAGTTCGAGGTTAATGTTCCGATAAACGCTTTAATTCTGCCAGTGTTACAACCAA	Amplify KanR with ORF38 flanking homologies
ORF39KanF	CAACATTTTTACTTTGGACTATAAACTGCGACTGAACGTTGCTCTTGTTGGCTAGTGCGTA	Amplify KanR with ORF39 flanking homologies
ORF39KanR	TTTTTAGACTGAACAATTATAGTACATTTATTGAAATGGATCTGCCAGTGTTACAACCAA	Amplify KanR with ORF39 flanking homologies
ORF40KanF	GTTTACACGTCCGGATTCAAGTCGCAACGCTGCTGACAAAGCTCTTGTTGGCTAGTGCGTA	Amplify KanR with ORF40 flanking homologies
ORF40KanR	CAGGGCTTTTATTGGGAGTGGGTATGTGGGCGTGGTGAAATCTGCCAGTGTTACAACCAA	Amplify KanR with ORF40 flanking homologies
ORF41KanF	GCTCGGCCGACTTGGTCTGTTTCACGCTTCCTTAAACAACGCTCTTGTTGGCTAGTGCGTA	Amplify KanR with ORF41 flanking homologies
ORF41KanR	GAAATGTAACTAGACTGGTTGTGTTTTTATTAACACCTGTTCTGCCAGTGTTACAACCAA	Amplify KanR with ORF41 flanking homologies
ORF42KanF	AAAACACAACCAGTCTAGTTACATTTCACGCGTCTTGTTTGCTCTTGTTGGCTAGTGCGTA	Amplify KanR with ORF42 flanking homologies
ORF42KanR	AACTTTGGTGGGGTAACTATATATCTTTCTATCGTTCCAGTCTGCCAGTGTTACAACCAA	Amplify KanR with ORF42 flanking homologies
ORF43KanF	TATTTCTTTTTTTTTCCAGTACAACCATATCCGGTGTATAGCTCTTGTTGGCTAGTGCGTA	Amplify KanR with ORF43 flanking homologies
ORF43KanR	TGAAGAGATTCTGGTGTTATGTTTTTATAGTGACACTAATTCTGCCAGTGTTACAACCAA	Amplify KanR with ORF43 flanking homologies
ORF44KanF	AAGTACCCGGGCGGCAATCCGCTAGACTGTTTTTCTGCTCGCTCTTGTTGGCTAGTGCGTA	Amplify KanR with ORF44 flanking homologies
ORF44KanR	GACATATACCTTTCCACAACGGCGGTTGAGTTAAGGTATATCTGCCAGTGTTACAACCAA	Amplify KanR with ORF44 flanking homologies
ORF45KanF	TACAGTAATATATTAAAGGTTAAATTTATAAAACACTCACGCTCTTGTTGGCTAGTGCGTA	Amplify KanR with ORF45 flanking homologies
ORF45KanR	GCTTACAAGAGATGAGGCGGATTCCGTGAATTGATGTTAATCTGCCAGTGTTACAACCAA	Amplify KanR with ORF45 flanking homologies

Table S1. Sequences of all primers used in VZV genomic functional profiling (cont.)

Primer Name	Primer Sequence (5`-3`)	Use
ORF46KanF	ATCATAAACATTTTCAGGGCCGCATTCATTCACTTTGGTCGCTCTTGTTGGCTAGTGCGTA	Amplify KanR with ORF46 flanking homologies
ORF46KanR	TCCATGCATGTTGCAGTTGACGGATTGGCCCATGGATGATTCTGCCAGTGTTACAACCAA	Amplify KanR with ORF46 flanking homologies
ORF47KanF	ATACTTGGTTTGGAGAAGACGACGAAGCGTTACTTACACAGCTCTTGTTGGCTAGTGCGTA	Amplify KanR with ORF47 flanking homologies
ORF47KanR	AGACCTCCCACACGGGCAATTTTTTTGGCTGGCTGGGGGCTCTGCCAGTGTTACAACCAA	Amplify KanR with ORF47 flanking homologies
ORF48KanF	GTCACGATAATTCAGAAATACGCTCGGATCACCCTTTATTGCTCTTGTTGGCTAGTGCGTA	Amplify KanR with ORF48 flanking homologies
ORF48KanR	ACATTCTCATCAAAGTCAAAGTCTTCAAAGAACTCTGAGTTCTGCCAGTGTTACAACCAA	Amplify KanR with ORF48 flanking homologies
ORF49KanF	CAGGAAACAACGCGATTACATCAGCATTGCGGTCATTGCGGCTCTTGTTGGCTAGTGCGTA	Amplify KanR with ORF49 flanking homologies
ORF49KanR	AACCATGATTTGATGTTTTTATTAGAACGTTTATCAGGGTTCTGCCAGTGTTACAACCAA	Amplify KanR with ORF49 flanking homologies
ORF50KanF	GTTTGTTTTATTGCTTGGGGGGTTACAAGTACAACCCACGGCTCTTGTTGGCTAGTGCGTA	Amplify KanR with ORF50 flanking homologies
ORF50KanR	CGCATAGACGGCGGCGTTGCTCTCCCCGGTGTTGGGAGACTCTGCCAGTGTTACAACCAA	Amplify KanR with ORF50 flanking homologies
ORF51KanF	TGAGACTTTTTCAGAACGCGGCCCCTTCTTTTGAGTTCCCGCTCTTGTTGGCTAGTGCGTA	Amplify KanR with ORF51 flanking homologies
ORF51KanR	GTAACCCCCCCTATGGATGTCCATATATGGTATTAATGGGTCTGCCAGTGTTACAACCAA	Amplify KanR with ORF51 flanking homologies
ORF52KanF	ACAAAGGGCCTCTAACAATGCACTGAACCACAACCAAGCTGCTCTTGTTGGCTAGTGCGTA	Amplify KanR with ORF52 flanking homologies
ORF52KanR	CTTTTATTTTAAGGATGGGTTGTTGCGGCGTGTTTTTTTGTCTGCCAGTGTTACAACCAA	Amplify KanR with ORF52 flanking homologies
ORF53KanF	AACACGCCGCAACAACCCATCCTTAAAATAAAAGGTTTATGCTCTTGTTGGCTAGTGCGTA	Amplify KanR with ORF53 flanking homologies
ORF53KanR	AAAGGACACCCCCCTGAGAGGTTACGGGTACACAAGGTAATCTGCCAGTGTTACAACCAA	Amplify KanR with ORF53 flanking homologies
ORF54KanF	CTCCCGCGCCTCGCATACGAATCTTGGTATTGCTTGTATTGCTCTTGTTGGCTAGTGCGTA	Amplify KanR with ORF54 flanking homologies
ORF54KanR	GAACTGTCTACAGAAATTGATCTTTTCATCGTGGTTTTCATCTGCCAGTGTTACAACCAA	Amplify KanR with ORF54 flanking homologies
ORF55KanF	TTATTAAAAAGAGACGTTATTTCGGCCATTGAAAACCACGGCTCTTGTTGGCTAGTGCGTA	Amplify KanR with ORF55 flanking homologies
ORF55KanR	TGAGAACAGCAAGCCCGCATGCGTGTTTTTATACAATGCTTCTGCCAGTGTTACAACCAA	Amplify KanR with ORF55 flanking homologies
ORF56KanF	AGGTTCTTAAAAATGAATCTAAACCCTCTACGGGAACGATGCTCTTGTTGGCTAGTGCGTA	Amplify KanR with ORF56 flanking homologies
ORF56KanR	ATGTAATCAGTTGGGATAAATGTTAGTCTTGAATCTGTCTTCTGCCAGTGTTACAACCAA	Amplify KanR with ORF56 flanking homologies
ORF57KanF	TTTATATTTAACGGCTTTTAATTTGAAGACACCTATCCTCGCTCTTGTTGGCTAGTGCGTA	Amplify KanR with ORF57 flanking homologies
ORF57KanR	ACTTTGACCGACCAACCAATTAATACTGAAAATAGCGGTCTCTGCCAGTGTTACAACCAA	Amplify KanR with ORF57 flanking homologies
ORF58KanF	ATGTTCCCCCGGCGTGGCAACGCTGGCATTTCCAAACACAGCTCTTGTTGGCTAGTGCGTA	Amplify KanR with ORF58 flanking homologies
ORF58KanR	GTGTTATATAACACTCCAATCGACCCTCTCGCGTACCATATCTGCCAGTGTTACAACCAA	Amplify KanR with ORF58 flanking homologies
ORF59KanF	TCCGAAAACATTATGGTACGCGAGAGGGTCGATTGGAGTGGCTCTTGTTGGCTAGTGCGTA	Amplify KanR with ORF59 flanking homologies
ORF59KanR	TGGGTAGACTCTACTCCATCTTCCACAAATATCCCGGAAATCTGCCAGTGTTACAACCAA	Amplify KanR with ORF59 flanking homologies
ORF60KanF	GGCCAAAACATAAATGTCCTTTGAATCCGATAGTTTCATTGCTCTTGTTGGCTAGTGCGTA	Amplify KanR with ORF60 flanking homologies
ORF60KanR	GGATACGACAACGTCGTAGTGAAGGGAAAACACAAGCGTCTCTGCCAGTGTTACAACCAA	Amplify KanR with ORF60 flanking homologies
ORF61KanF	CCGTGGATGTTTAAGGCATTTCCTTCCCCCTCCCAACAAAGCTCTTGTTGGCTAGTGCGTA	Amplify KanR with ORF61 flanking homologies
ORF61KanR	GTTTGGATGCCCGGACATTAGAATACAGCCAGTTGTTACCTCTGCCAGTGTTACAACCAA	Amplify KanR with ORF61 flanking homologies
ORF62/71KanF	CGCGCGCCAGTGGCGCTCACGAGAAAAGGAGGGGACTCCGGCTCTTGTTGGCTAGTGCGTA	Amplify KanR with ORF62/71 flanking homologies
ORF62/71KanR	GTTTGGAAAACTCGGGTACGTCTAAATTCACCCCAGTGCGTCTGCCAGTGTTACAACCAA	Amplify KanR with ORF62/71 flanking homologies
ORF63/70KanF	AGTCGGTTTTAAGGGAAAAGGTTACTACGGCCCCAAGGACGCTCTTGTTGGCTAGTGCGTA	Amplify KanR with ORF63/70 flanking homologies
ORF63/70KanR	AAAAAGACACGAGCCAAACCATTGTATTTATTTATAAAGATCTGCCAGTGTTACAACCAA	Amplify KanR with ORF63/70 flanking homologies
ORF64/69KanF	TCTCCATTAACCCACGGGGTCCCCACACGGGCCGTGTGGTGCTCTTGTTGGCTAGTGCGTA	Amplify KanR with ORF64/69 flanking homologies
ORF64/69KanR	TTTAAATTTATTTTTTTTTTCTATATAAAGGGATGGGGTGTCTGCCAGTGTTACAACCAA	Amplify KanR with ORF64/69 flanking homologies
ORF65KanF	CTTCCGTATCCGTAGATTCCGAGTCCTCGAAATCGTCCACGCTCTTGTTGGCTAGTGCGTA	Amplify KanR with ORF65 flanking homologies
ORF65KanR	ACTTTTAAACTAGCCTGCTGGCCTTACAATCTGGATTTCTTCTGCCAGTGTTACAACCAA	Amplify KanR with ORF65 flanking homologies
ORF66KanF	TCATTAACACTATCAACATAAGTCGGGGTATACAAGTATACTCTTGTTGGCTAGTGCGTA	Amplify KanR with ORF66 flanking homologies
ORF66KanR	CACAATTTATACAATATTTTATTAACAGGCTTAATGAATTTCTGCCAGTGTTACAACCAA	Amplify KanR with ORF66 flanking homologies
ORF67KanF	CGGTGTAGTTGGGTATACTGGTGCGCCTCATTTAATCGCGGCTCTTGTTGGCTAGTGCGTA	Amplify KanR with ORF67 flanking homologies
ORF67KanR	GCATATAGTTTATTTAATATAAAATCCGGGATAATTAGTTTCTGCCAGTGTTACAACCAA	Amplify KanR with ORF67 flanking homologies
ORF68KanF	CGTGGTTTTAAGTGACTATATTCCGAGGGTCGCCTGTAATGCTCTTGTTGGCTAGTGCGTA	Amplify KanR with ORF68 flanking homologies
ORF68KanR	TATTTAAATTTACACGCTCGGCGTTGCCCCGGTTCGGTGATCTGCCAGTGTTACAACCAA	Amplify KanR with ORF68 flanking homologies
ORF62/71AmpF	CGCGCGCCAGTGGCGCTCACGAGAAAAGGAGGGGACTCCGGAGTAAACTTGGTCTGACAGTTACC	Amplify AmpR with ORF62/71 flanking homologies
ORF62/71AmpR	GTTTGGAAAACTCGGGTACGTCTAAATTCACCCCAGTGCGTTAATGCGCCGCTACAGGGC	Amplify AmpR with ORF62/71 flanking homologies

Table S1. Sequences of all primers used in VZV genomic functional profiling (cont.)

Primer Name	Primer Sequence (5`-3`)	Use
ORF63/70AmpF	AGTCGGTTTTAAGGGAAAAGGTTACTACGGCCCCAAGGACGAGTAAACTTGGTCTGACAGTTACC	Amplify AmpR with ORF63/70 flanking homologies
ORF63/70AmpR	AAAAAGACACGAGCCAAACCATTGTATTTATTTATAAAGATTAATGCGCCGCTACAGGGC	Amplify AmpR with ORF63/70 flanking homologies
ORF64/69AmpF	TCTCCATTAACCCACGGGGTCCCCACACGGGCCGTGTGGTGAGTAAACTTGGTCTGACAGTTACC	Amplify AmpR with ORF64/69 flanking homologies
ORF64/69AmpR	TTTAAATTTATTTTTTTTTTCTATATAAAGGGATGGGGTGTTAATGCGCCGCTACAGGGC	Amplify AmpR with ORF64/69 flanking homologies
ORF0F	ATGGCGACCGTGCACTACTC	Amplify ORF0
ORF0R	TCATGTAGTTGAGTTGGGAGGTTC	Amplify ORF0
ORF1F	TTATTCTCGCTTGCAGCTTGTCG	Amplify ORF1
ORF1R	ATGTCCAGGGTATCGGAGTATG	Amplify ORF1
ORF2F	ATGCATGTAATTTCTGAGACACTTGCATA	Amplify ORF2
ORF2R	TTACATCAATACGCCCTCCGTAG	Amplify ORF2
ORF3F	TCATAGTCCGCCGACAGCC	Amplify ORF3
ORF3R	ATGGATACAACGGGAGCTTCC	Amplify ORF3
ORF4F	TTAGCAGTTAAAGGTACTACACTTAAAATATTTA	Amplify ORF4
ORF4R	ATGGCCTCTGCTTCAATTCCAAC	Amplify ORF4
ORF5F	TTAATGCTTCTGGGAGTTTTCACTTTC	Amplify ORF5
ORF5R	ATGCAGGCTTTAGGAATCAAGACAG	Amplify ORF5
ORF6F	TTAACTCGAAGTTAAATTTGGATAATTAGGTA	Amplify ORF6
ORF6R	ATGGATAAATCCTCCAAACCGACGA	Amplify ORF6
ORF7F	ATGCAGACGGTGTGTGCCAG	Amplify ORF7
ORF7R	TTATACAAGCATAACATGGGATTTCTTGAT	Amplify ORF7
ORF8/9AF	TTAATGTTTTAGTAGAAAATCGACATCGTTTG	Amplify ORF8/9A
ORF8/9AR	TTACCACGTGCTGCGTAATACAGAA	Amplify ORF8/9A
ORF9F	ATGGCATCTTCCGACGGTGAC	Amplify ORF9
ORF9R	CTATTTTCGCGTATCAGTTCTTGATG	Amplify ORF9
ORF10F	ATGCAGTCGGGTCATTATAACCG	Amplify ORF10
ORF10R	TTAATATTTTCGTAGTAAATGCATGGCTAC	Amplify ORF10
ORF11F	ATGTTTTCTCGGTTTGCGCGTTC	Amplify ORF11
ORF11R	TTAATGATGACTCTTAGGCGTATTTTTCC	Amplify ORF11
ORF12F	ATGGGAGACTTGTCATGTTGGACAA	Amplify ORF12
ORF12R	TTAAAGAGCCATTTCCATTTTTAGGGG	Amplify ORF12
ORF13F	TTATGAACAGCAACGGATGCATAGG	Amplify ORF13
ORF13R	ATGAAGCGGATACAAATAAATTTAATTTTAACGAT	Amplify ORF13
ORF14F	TTATGAACAGCAACGGATGCATAGG	Amplify ORF14
ORF14R	ATGAAGCGGATACAAATAAATTTAATTTTAACGAT	Amplify ORF14
ORF15F	TTACGATACATATGTACCACATAGATAGC	Amplify ORF15
ORF15R	ATGGCCGTGAATGGTGAAAGAGC	Amplify ORF15
ORF16F	TTATTTAACTGTACATATTACGTCAGATTCAC	Amplify ORF16
ORF16R	ATGGATTTGAGGTCGCGTACAGA	Amplify ORF16
ORF17F	ATGGGGCTCTTTGGACTGACAC	Amplify ORF17
ORF17R	TTAATTCCAATATTTTGTTAATACAGTGTTTAGTG	Amplify ORF17
ORF18F	TTATAAATCGTTTATCACTGTGCCCG	Amplify ORF18
ORF18R	ATGGATCAGAAAGATTGCAGTCATTTTTTT	Amplify ORF18
ORF19F	TTATAAAGCACAACTGGTACAGGTTAATT	Amplify ORF19
ORF19R	ATGGAGTTCAAAAGAATTTTTAATACGGTTC	Amplify ORF19
ORF20F	TTAATAATAACATTCGTTCCATGTATTTGTACC	Amplify ORF20
ORF20R	ATGGGGAGTCAACCAACCAACTC	Amplify ORF20

Table S1. Sequences of all primers used in VZV genomic functional profiling (cont.)

Primer Name	Primer Sequence (5`-3`)	Use
ORF21F	ATGGAAGAACCAATTTGTTATGATACACAAA	Amplify ORF21
ORF21R	TTAAGGGTCACTCCCACTTGTATTC	Amplify ORF21
ORF22F	ATGGATATAATTCCGCCTATAGCTGTC	Amplify ORF22
ORF22R	TTATATATATGTTCCATCTAATAACAATTTAACATACC	Amplify ORF22
ORF23F	TTACACCCTACGACTTCTTGAAGC	Amplify ORF23
ORF23R	ATGACACAACCCGCATCGTCTC	Amplify ORF23
ORF24F	TTATTTCCAGAAAAGCACCGCCC	Amplify ORF24
ORF24R	ATGTCACGGAGAACGTATGTACG	Amplify ORF24
ORF25F	TTAAGCATCCTTCAATATTTCATGCAAATCT	Amplify ORF25
ORF25R	ATGTACGAATCGGAAAATGCGTCG	Amplify ORF25
ORF26F	ATGGATCGGGTAGAATCAGAAGAAC	Amplify ORF26
ORF26R	CTAGACATACTTCGATAGGGTGTGT	Amplify ORF26
ORF27F	ATGCATTTAAAGCCTACCAGATTTTTCC	Amplify ORF27
ORF27R	TCACCGAGGAGGAACAAAGTCAT	Amplify ORF27
ORF28F	TTAACTTTGATGGAGAATTGCTTTTGGAATA	Amplify ORF28
ORF28R	ATGGCGATCAGAACGGGGTTTTG	Amplify ORF28
ORF29F	ATGGAAAATACTCAGAAGACTGTGACA	Amplify ORF29
ORF29R	TTAAATCATTTCCATTGTAATGTTCCCATGT	Amplify ORF29
ORF30F	ATGGAATTGGATATTAATCGAACATTGTTGG	Amplify ORF30
ORF30R	TTATGAAAACGCCGGGTCCGTTG	Amplify ORF30
ORF31F	ATGTTTGTTACGGCGGTTGTGTC	Amplify ORF31
ORF31R	TTACACCCCCGTTACATTCTCGG	Amplify ORF31
ORF32F	ATGGAATCGTCTAACATTAACGCGC	Amplify ORF32
ORF32R	TTAATCGGTGTCAGAATCTTCATCCC	Amplify ORF32
ORF33.5/33F	TTAACACCGCCCCACCATCATC	Amplify ORF33.5/33
ORF33.5/33R	ATGGCTGCTGAAGCTGACGAAG	Amplify ORF33.5/33
ORF34F	TCACGGTGTGGAGGCAAACTG	Amplify ORF34
ORF34R	ATGACGGCGAGATATGGGTTCG	Amplify ORF34
ORF35F	TTACCCATGGGAAAACATCCCGG	Amplify ORF35
ORF35R	ATGTCCGCTAGTCGAATTCGGG	Amplify ORF35
ORF36F	ATGTCAACGGATAAAACCGATGTAAAAATG	Amplify ORF36
ORF36R	TTAGGAAGTGTTGTCCTGAACGG	Amplify ORF36
ORF37F	ATGTTTGCGCTAGTTTTAGCGGTG	Amplify ORF37
ORF37R	TTATGTCAGAGGTATTTTATTATATTCTCGAAG	Amplify ORF37
ORF38F	CTACCTTTGGGTTTTTTTCCCGTC	Amplify ORF38
ORF38R	ATGGAATTTCCATATCATTCAACCGTATC	Amplify ORF38
ORF39F	ATGAACCCACCCCAAGCCC	Amplify ORF39
ORF39R	CTAAAACGAAATAGATGTTTTTAACATAACACG	Amplify ORF39
ORF40F	ATGACAACGGTTTCATGTCCCGC	Amplify ORF40
ORF40R	TTATCGCGGAAGAGGAAGACATC	Amplify ORF40
ORF41F	ATGGCTATGCCATTTGAGATAGAGG	Amplify ORF41
ORF41R	TTACACTTGAATCACGGCCGTGC	Amplify ORF41
ORF42F	TTATTTAATAGGCATAAACACGGAATCCG	Amplify ORF42
ORF42R	GGTATCCGAGGTCAAGATTTTAATCTT	Amplify ORF42
ORF43F	ATGGAAGCCCATTTGGCAAATGAAAC	Amplify ORF43
ORF43R	TTATTTATGGGGGTTGGGAATAGAGAA	Amplify ORF43

Table S1. Sequences of all primers used in VZV genomic functional profiling (cont.)

Primer Name	Primer Sequence (5`-3`)	Use
ORF44F	ATGGAATTACAACGCATATTTCCGCTG	Amplify ORF44
ORF44R	CTAGGTGGTTGTAGGTTCCGG	Amplify ORF44
ORF45F	GTTTGTGTTGTGACTTGACGCGAA	Amplify ORF45
ORF45R	ATGTCATTGATAATGTTTGGTCGTACG	Amplify ORF45
ORF46F	ATGTCAGGCCACACTCCAACC	Amplify ORF46
ORF46R	TTACACATCCGTGTGTGGGGTTG	Amplify ORF46
ORF47F	ATGGATGCTGACGACACACCC	Amplify ORF47
ORF47R	TTATGTCGATCCTATCCAATCCCGA	Amplify ORF47
ORF48F	ATGGCACGATCGGGATTGGATAG	Amplify ORF48
ORF48R	TCAAAGCAACGGTTTCTCCGTTG	Amplify ORF48
ORF49F	ATGGGACAATCTTCATCCAGCGG	Amplify ORF49
ORF49R	TTAACATTTTGCGCATTTGGAATGGGA	Amplify ORF49
ORF50F	CTACTCCCACCCACTGTTTGATC	Amplify ORF50
ORF50R	ATGGGAACTCAAAAGAAGGGGCC	Amplify ORF50
ORF51F	ATGTCTCCCAACACCGGGGA	Amplify ORF51
ORF51R	TTATAAACTTTCAAAATTTACCGCCCCG	Amplify ORF51
ORF52F	ATGGACGCAACGCAGATTACCTT	Amplify ORF52
ORF52R	TCATAAAAACAAGAAGTTATATGAAGCAAGG	Amplify ORF52
ORF53F	TTACTTTACAACCCGTGGTGAATTTTTATAC	Amplify ORF53
ORF53R	ATGCAGCGGATTCGACCTTACTG	Amplify ORF53
ORF54F	CTAAGATCTTCGATCACGTCGCT	Amplify ORF54
ORF54R	ATGGCCGAAATAACGTCTCTTTTTAATAAC	Amplify ORF54
ORF55F	ATGAAAAGATCAATTTCTGTAGACAGTTCTT	Amplify ORF55
ORF55R	TTAATACACAACGTGTACGTTGGGATC	Amplify ORF55
ORF56F	ATGAAAAATCCGCAGAAATTAGCGATCA	Amplify ORF56
ORF56R	TTACGCGTTTGCGGCGTCC	Amplify ORF56
ORF57F	TTAACGTTGAGGAGCCTTGCAGG	Amplify ORF57
ORF57R	ATGGACGTACGAGAACGTAATGTGT	Amplify ORF57
ORF58F	TTACGTTCTCGTACGTCCATGAC	Amplify ORF58
ORF58R	ATGTTTTCGGAGTTGCCTCCTTC	Amplify ORF58
ORF59F	TTATATAACACTCCAATCGATCTCGGG	Amplify ORF59
ORF59R	ATGGATGTGTCTGGGGAGCC	Amplify ORF59
ORF60F	TCATTGGCATACGCGTTGGAACAAA	Amplify ORF60
ORF60R	ATGGCATCACATAAATGGTTACTGCAG	Amplify ORF60
ORF61F	CTAGGACTTCTTCATCTTGTTTGGAATA	Amplify ORF61
ORF61R	ATGGATACCATATTAGCGGGCGG	Amplify ORF61
ORF62/71F	TCACCCCCGACTCTGCGG	Amplify ORF62/71
ORF62/71R	ATGGATACGCCGCCGATGCA	Amplify ORF62/71
ORF63/70F	ATGTTTTGCACCTCACCGGCTAC	Amplify ORF63/70
ORF63/70R	CGGTATATCATGCCGGCGC	Amplify ORF63/70
ORF64/69F	ATGAATCTCTGCGGATCCCGC	Amplify ORF64/69
ORF64/69R	TCAGGATCTCTCGTAGGTTCTTG	Amplify ORF64/69
ORF65F	TTATCCAACAAATTGTGACGTTATATATCC	Amplify ORF65
ORF65R	ATGGCCGGACAAAACACCATGGA	Amplify ORF65
ORF66F	ATGAACGACGTTGATGCAACAGACA	Amplify ORF66
ORF66R	TTAATCTCCAACTTCCATTGGATTTGGAT	Amplify ORF66
ORF67F	ATGTTTTTAATCCAATGTTTGATATCG	Amplify ORF67
ORF67R	ACATTCCGTTGTAAACCCGTT	Amplify ORF67

Table S1. Sequences of all primers used in VZV genomic functional profiling (cont.)

Primer Name	Primer Sequence (5`-3`)	Use
ORF68F	ATGGGGACAGTTAATAAACCTGTGG	Amplify ORF68
ORF68R	TCACCGGGTCTTATCTATATACACC	Amplify ORF68
ORF8PKanF	TATAAAATTAAAACATTGCTGGCTGGCGTGGTTATTACATTAGCTAGCTAGGCTCTTGTTGGCTAGTGCGTA	Amplify KanR cassette targeting partial ORF8
ORF8PKanR	GAATCGGTGTTTAAAAGATACAATTTTAATAGAAGTTCAACTAGCTAGCTATCTGCCAGTGTTACAACCAA	Amplify KanR cassette targeting partial ORF8
ORF9APKanF	ACTTTGCAGAACGGAATTTTTGGCATGCCAGCTGTTCGTAGCTAGCTAGGCTCTTGTTGGCTAGTGCGTA	Amplify KanR cassette targeting partial ORF9A
ORF9APKanR	CCCTCTTATACACGCCTGCCCCTTTTATAGGCAAACGGGTCTAGCTAGCTATCTGCCAGTGTTACAACCAA	Amplify KanR cassette targeting partial ORF9A
ORF25PKanF	CCCCCTGACGACTTATTAATGCGTTTATTTTCCCCATGTATAGCTAGCTAGGCTCTTGTTGGCTAGTGCGTA	Amplify KanR cassette targeting partial ORF25
ORF25PKanR	TCGATGCACTGGAGTCAAAGTATTTTCCTGCTGATAGCACTAGCTAGCTATCTGCCAGTGTTACAACCAA	Amplify KanR cassette targeting partial ORF25
ORF26PKanF	TTATTTTCAGAATTGTTATTTGCCTCCCACTTAATAAATGTAGCTAGCTAGGCTCTTGTTGGCTAGTGCGTA	Amplify KanR cassette targeting partial ORF26
ORF26PKanR	TCGAAGCATAAGTCCTCCATCTCGTATGAATGCGGCATCTAGCTAGCTATCTGCCAGTGTTACAACCAA	Amplify KanR cassette targeting partial ORF26
ORF27PKanF	CCAGAACGCCATGCCGCCGTCAAACCATTCGAGGAAATAGCTAGCTAGGCTCTTGTTGGCTAGTGCGTA	Amplify KanR cassette targeting partial ORF27
ORF27PKanR	TCTCTGTTTGTTGTTCTGGGTTGCGACATACCGACAGTACTAGCTAGCTATCTGCCAGTGTTACAACCAA	Amplify KanR cassette targeting partial ORF27
ORF28PKanF	AAAGCCTGCCGCCTGCAAGCGGTTTAGCATTTTAACGTTTAGCTAGCTAGGCTCTTGTTGGCTAGTGCGTA	Amplify KanR cassette targeting partial ORF28
ORF28PKanR	TTTATGTTAACGATTGATAATCTTGTAAAAAGCCAGTGTTCTAGCTAGCTATCTGCCAGTGTTACAACCAA	Amplify KanR cassette targeting partial ORF28
ORF46PKanF	ATCATAAACATTTTCAGGGCCGCATTCATTCACTTTGGTCTAGCTAGCTAGGCTCTTGTTGGCTAGTGCGTA	Amplify KanR cassette targeting partial ORF46
ORF46PKanR	GATTTTCGTGTTCGATGTTCGGTCCTACCGCGTCTTCCAGCTAGCTAGCTATCTGCCAGTGTTACAACCAA	Amplify KanR cassette targeting partial ORF46
ORF47PKanF	ATCACAACAGACAAGCTTATTTTTATTAAAGCACGGCTTATAGCTAGCTAGGCTCTTGTTGGCTAGTGCGTA	Amplify KanR cassette targeting partial ORF47
ORF47PKanR	ACGACGTAACAACAGTGGCTGGGTATAATGAGGCCGGACTAGCTAGCTATCTGCCAGTGTTACAACCAA	Amplify KanR cassette targeting partial ORF47
ORF48PKanF	TTTGTAAAAACGGATATTAACACGATTAACGTTGAACACTAGCTAGCTAGGCTCTTGTTGGCTAGTGCGTA	Amplify KanR cassette targeting partial ORF48
ORF48PKanR	ATTTTTGTCACTCTAGGCACATCAACTTCGATAGGCGTTAGCTAGCTAGCTATCTGCCAGTGTTACAACCAA	Amplify KanR cassette targeting partial ORF48
ORF49PKanF	ACGCCGATAAATCCACACAACGCCGCCCACGAGTGATCTAGCTAGCTAGGCTCTTGTTGGCTAGTGCGTA	Amplify KanR cassette targeting partial ORF49
ORF49PKanR	AACCATGATTTGATGTTTTTATTAGAACGTTTATCAGGGTTTCTAGCTAGCTATCTGCCAGTGTTACAACCAA	Amplify KanR cassette targeting partial ORF49
ORF50PKanF	GTTTGTTTTATTGCTTGGGGGGTTACAAGTACAACCCACGTAGCTAGCTAGGCTCTTGTTGGCTAGTGCGTA	Amplify KanR cassette targeting partial ORF50
ORF50PKanR	CCGTACGACACCACGACACCCGAGGTGGAAGCGTTAGACTAGCTAGCTATCTGCCAGTGTTACAACCAA	Amplify KanR cassette targeting partial ORF50
ORF54PKanF	GATAGGTTTTTATATCCAACATGCATGTATTGGTTATTTATTTAGCTAGCTAGGCTCTTGTTGGCTAGTGCGTA	Amplify KanR cassette targeting partial ORF54
ORF54PKanR	TCCGGTAGTGAAGAAAAAAGGATAGCAAGTTCTGTTTCTACTAGCTAGCTATCTGCCAGTGTTACAACCAA	Amplify KanR cassette targeting partial ORF54
ORF56PKanF	TTTGTCTTAAATACACCCGCCATGAGCATCTCTGGACCCCTAGCTAGCTAGGCTCTTGTTGGCTAGTGCGTA	Amplify KanR cassette targeting partial ORF56
ORF56PKanR	AGACAGATTCAAGACTAACATTTATCCCAACTGATTACATCTAGCTAGCTATCTGCCAGTGTTACAACCAA	Amplify KanR cassette targeting partial ORF56
ORF59PKanF	ATCTCGGGTTCGCCTTTACGCGTAAAATACTCATTGGCTTTAGCTAGCTAGGCTCTTGTTGGCTAGTGCGTA	Amplify KanR cassette targeting partial ORF59
ORF59PKanR	CATCCGGTTACCAAAAAAACCCGCAAGCGACCCCGCGGCTAGCTAGCTATCTGCCAGTGTTACAACCAA	Amplify KanR cassette targeting partial ORF59
ORF60PKanF	GGGCATCCAACAAAGCATCGCGTATGTCCCCGCTTTTATGTAGCTAGCTAGGCTCTTGTTGGCTAGTGCGTA	Amplify KanR cassette targeting partial ORF60
ORF60PKanR	AGTCGAAGATCTTCATGTAGTTGAGTTGGGAGGTTC	Amplify KanR cassette targeting partial ORF60
VZVORF0ZeoF	AACCCGCGCCTTTTGCGTCCACCCCTCGTTTACTGCTCGGATGGCGACCGTGCACTACTC	Amplify ORF0-zeoR cassette
VZVORF0ZeoR	GCAAGCGAGAATAAATACCTTCCCCTTCCGGACAGTAGTTGGATGGATCCATAACTTCGT	Amplify ORF0-zeoR cassette
VZVORF0SZeoF	AACCCGCGCCTTTTGCGTCCACCCCTCGTTTACTGCTCGGATGGCGACCGTGCACTAATC	Amplify ORF0-zeoR cassette with a stop codon
VZVORF0SZeoR	GCAAGCGAGAATAAATACCTTCCCCTTCCGGACAGTAGTTGGATGGATCCATAACTTCGT	Amplify ORF0-zeoR cassette with a stop codon
VZVORF4SpeIF	AGTCGAACTAGTTTAGCAGTTAAAGGTACTACACTTAAAATATTTA	Directionally clone ORF4 into pGEM-lox-zeo
VZVORF4NdeIR	AGTCGACATATGATGGCCTCTGCTTCAATTCCAAC	Directionally clone ORF4 into pGEM-lox-zeo
VZVORF4ZeoF	TTAGTATGTTTTGACAAGCATGAAAAAGGTATTTTTTATTGGATGGATCCATAACTTCGT	Amplify ORF4-zeoR cassette
VZVORF4ZeoR	AGGCAACTGCAAACACGCAATTGTCAGATATTTTGCAGCCATGGCCTCTGCTTCAATTCCAAC	Amplify ORF4-zeoR cassette
VZVORF7NotIF	AGTCGAGCGGCCGCATGCAGACGGTGTGTGCCAG	Directionally clone ORF7 into pGEM-lox-zeo
VZVORF7BglIIR	AGTCGAAGATCTTTATACAAGCATAACATGGGATTTCTTGAT	Directionally clone ORF7 into pGEM-lox-zeo
VZVORF7ZeoF	GATTTATCCATAGTTCAATACGTTGGAAAGCCAGTCAATCATGCAGACGGTGTGTGCCAG	Amplify ORF7-zeoR cassette
VZVORF7ZeoR	AAACATACACCAGAAACGTTTTTAGTTTTTATTTCAATATGGATGGATCCATAACTTCGT	Amplify ORF7-zeoR cassette
VZVORF7SZeoF	GATTTATCCATAGTTCAATACGTTGGAAAGCCAGTCAATCATGCAGACGGTGTGAGCCAG	Amplify ORF7-zeoR cassette with a stop codon
VZVORF7SZeoR	AAACATACACCAGAAACGTTTTTAGTTTTTATTTCAATATGGATGGATCCATAACTTCGT	Amplify ORF7-zeoR cassette with a stop codon
VZVORF9NdeIF	AGTCGACATATGATGGCATCTTCCGACGGTGAC	Directionally clone ORF9 into pGEM-lox-zeo
VZVORF9SpeIR	AGTCGAACTAGTCTATTTTCGCGTATCAGTTCTTGATG	Directionally clone ORF9 into pGEM-lox-zeo

Table S1. Sequences of all primers used in VZV genomic functional profiling (cont.)

Primer Name	Primer Sequence (5`-3`)	Use
VZVORF9ZeoF	CGTGTTTGGATATTTCACGACCCTATCGTTTATTTACGTAATGGCATCTTCCGACGGTGAC	Amplify ORF9-zeoR cassette
VZVORF9ZeoR	TACATAATACCGGGTAAACCGTTACTGCGTAATTATATCCGGATGGATCCATAACTTCGT	Amplify ORF9-zeoR cassette
VZVORF10NdeIF	AGTCGACATATGATGGAGTGTAATTTAGGAACCGAAC	Directionally clone ORF10 into pGEM-lox-zeo
VZVORF10SpeIR	AGTCGAACTAGTTTAACGCGTTAAAAACCCACAC	Directionally clone ORF10 into pGEM-lox-zeo
VZVORF10ZeoF	GGGAATCGCTTATTTAAACTAAAGATTTTACTCTATAAGTATGGAGTGTAATTTAGGAACCGAAC	Amplify ORF10-zeoR cassette
VZVORF10ZeoR	CGTTTTCGTAATTTATTTACACCCTCTACCCCAATGACGTGGATGGATCCATAACTTCGT	Amplify ORF10-zeoR cassette
KanF	GCTCTTGTTGGCTAGTGCGTA	Amplify KanR cassette
KanR	TCTGCCAGTGTTACAACCAA	Amplify KanR cassette
AmpF	GAGTAAACTTGGTCTGACAGTTACC	Amplify AmpR cassette
AmpR	TTAATGCGCCGCTACAGGGC	Amplify AmpR cassette
VZVORF5SpeIF	AGTCGAACTAGTTTAATGCTTCTGGGAGTTTTCACTTTC	Directionally clone ORF5 into pGEM-lox-zeo
VZVORF5NdeIR	AGTCGACATATGATGCAGGCTTTAGGAATCAAGACAG	Directionally clone ORF5 into pGEM-lox-zeo
VZVORF5ZeoF	GGCTCACCCAACCCCGCAATGGGCGTGTTTAGTCACATGAGGATGGATCCATAACTTCGT	Amplify ORF5-zeoR cassette
VZVORF5ZeoR	TGATACTACATCGTGCTTGAATTGCCATCTTCCACGGGTCATGCAGGCTTTAGGAATCAAGACAG	Amplify ORF5-zeoR cassette
