# Supplementary material for: Genome-Wide Mutagenesis Reveals That ORF7 Is a Novel VZV Skin-Tropic Factor
Source: PLoS Pathog. 2010 Jul 1;6(7):e1000971. doi: 10.1371/journal.ppat.1000971 (PMC2895648; doi:10.1371/journal.ppat.1000971)
Supplement: Table S2 — Description of Overlapping VZV ORFs. (0.05 MB PDF) [file ppat.1000971.s002.pdf]

Table S2. Description of Overlapping VZV ORFs

Gene (Deletion)	Overlapping Gene(s)	Distance between Deletion and Overlap	Overlap (# bp)	Percentage Overlap	Position	Category
ORF8	ORF9A	92 bp	25 bp	2.1%	Upstream	Dispensable
ORF9A	ORF8	94 bp	25 bp	9.5%	Upstream	Essential
ORF25	ORF26	97 bp	112 bp	23.8%	Upstream	Essential
ORF26	ORF25, ORF27	78 bp, 21 bp	185 bp	10.5%	Both	Essential
ORF27	ORF26, ORF28	88 bp, 92 bp	149 bp	15.9%	Both	Essential
ORF28	ORF27	93 bp	76 bp	2.1%	Downstream	Essential
ORF46	ORF47	81 bp	150 bp	25.0%	Downstream	Essential
ORF47	ORF46, ORF48	93 bp, 87 bp	183 bp	11.9%	Both	Skin-tropic
ORF48	ORF47, ORF49	91 bp, 92 bp	129 bp	7.8%	Both	Essential
ORF49	ORF48	89 bp	96 bp	39.2%	Upstream	Growth-Defect
ORF50	ORF51	81 bp	1 bp	0.1%	Upstream	Essential
ORF54	ORF53	91 bp	175 bp	7.6%	Downstream	Essential
ORF59	ORF60	81 bp	49 bp	5.3%	Upstream	Dispensable
ORF60	ORF59	92 bp	49 bp	10.2%	Downstream	Essential

ORF60 was originally designed as a partial deletion primer, but it has been removed from this list because the most recent GenBank Annotation for VZV (Accession Number: NC_001348) no longer shows ORF60 overlapping ORF55. We did not make partial deletion mutants for ORF51 or ORF53, so the phenotype for these genes may be due to deletion of their respective adjacent genes, ORF50 and ORF54 respectively. This potential influence has been noted in Figure 1.
