# Supplementary material for: Genome-Wide Mutagenesis Reveals That ORF7 Is a Novel VZV Skin-Tropic Factor
Source: PLoS Pathog. 2010 Jul 1;6(7):e1000971. doi: 10.1371/journal.ppat.1000971 (PMC2895648; doi:10.1371/journal.ppat.1000971)
Supplement: Table S3 — Essentiality of Essential VZV Homologous in HSV and CMV. (0.05 MB PDF) [file ppat.1000971.s003.pdf]

Table S3. Essentiality of Essential VZV Homologous in HSV and CMV

Gene	HSV Homolog	CMV Homolog
ORF4	UL54	None
ORF5	UL53	None
ORF6	UL52	UL70
ORF9A	UL49A	None
ORF9	UL49	None
ORF16	UL42	None
ORF17	UL41	None
ORF20	UL38	None
ORF21	UL37	None
ORF22	UL36	UL48
ORF24	UL34	None
ORF25	UL33	UL51
ORF26	UL32	UL52
ORF27	UL31	UL53
ORF28	UL30	UL54
ORF29	UL29	UL57
ORF30	UL28	UL56
ORF31	UL27	UL55
ORF33	UL26	UL80
ORF33.5	UL26.5	None
ORF34	UL25	UL77
ORF37	UL22	None
ORF38	UL21	None
ORF39	UL20	None
ORF40	UL19	UL86
ORF41	UL18	UL85
ORF42	UL15	UL89
ORF43	UL17	None
ORF44	UL16	None
ORF46	UL14	None
ORF48	UL12	UL98
ORF50	UL10	UL100
ORF51	UL9	None
ORF52	UL8	None
ORF53	UL7	None
ORF54	UL6	UL104
ORF55	UL5	UL105
ORF56	UL4	None
ORF60	UL1	None
ORF61	ICP0	None
ORF62/71	ICP4	None
ORF63/70	US1	None
ORF66	US3	None
ORF68	US8	None

NOTE: essential genes appear in red; nonessential genes appear in black; blue represents genes with tissue specificity and green denotes a gene whose essentiality has not been reported.
