# Supplementary material for: Genome-Wide Mutagenesis Reveals That ORF7 Is a Novel VZV Skin-Tropic Factor
Source: PLoS Pathog. 2010 Jul 1;6(7):e1000971. doi: 10.1371/journal.ppat.1000971 (PMC2895648; doi:10.1371/journal.ppat.1000971)
Supplement: Text S1 — Supplementary Materials. (0.03 MB DOC) [file ppat.1000971.s004.doc]

# Supplementary Materials

**Generating linear ORF targeting cassettes**

All primers were synthesized by Sigma-Genosys (Woodlands, TX) and stored in TE buffer (100uM). Primer sequences are listed in Supplementary Table 1. HotStar Taq DNA polymerase (Qiagen, Valencia, CA) was used for general PCR reactions and Platinum Taq DNA polymerase (Invitrogen, Carlsbad, CA) was used for hi-fidelity PCR, both following standard PCR conditions. PCR purification was carried out using a PCR purification kit (Qiagen, Valencia, CA) following the manufacturer’s protocol. The amplified linear DNAs were suspended in sterile ddH2O and were quantified by spectroscopy (NanoDrop Technologies, Inc., Wilmington, DE). In order to achieve optimum results, the final concentration of the linear DNA cassette for the subsequent electroporation was adjusted to at least 100ng/ul. DpnI (New England Biolabs, Inc., Ipswich, MA) treatment following PCR was carried out in order to eliminate circular template DNA. Electroporation was carried out at 1.6kV, 200ohm and 25uF on a Gene Pulser II electroporator (Bio-Rad, Hercules, CA). Two ul of concentrated linear DNA cassette (>200ng) was used for each reaction.

**Induction of lambda recombination system and preparation of electroporation-competent DY380**

DY380 cells were grown at 32°C until the OD_600nm_ measurement reached 0.5. The temperature of the culture was increased to 42°C by placing the flask into a 42°C water bath and shaking it vigorously for 10–15 min. The success of the homologous recombination hinges on both the precise adjustment of the temperature to 42°C and the subsequent maintenance of that temperature. Too much recombination system activity is detrimental to E. coli and will harm the integrity of the BAC DNA. On the other hand, inadequate induction of the recombination system in DY 380 will lead to inefficient recombination. The ten to fifteen minute time span might need to be adjusted in order to achieve optimized efficiency of homologous recombination. (After this point, every step needs to be carried out at a low temperature (0-4°C); all reagents, the centrifuge rotor, and the glassware need to be pre-chilled.) The culture was immediately transferred to ice-water slurry for 30 min, then pelleted, washed with ice-cold sterile ddH_2_O, and repelleted. Ten percent glycerol (1% of original volume of culture) was used to resuspend cells, and a 40ul aliquot (>1X10^10^ cells) was used for each electroporation reaction.

**Antibiotics and selection**

The bacteria were transferred to 1ml of LB medium immediately after electroporation and recovered at 32°C for 1hr before plating. All antibiotics were obtained from Sigma (St. Louis, MO). LB plates containing the proper antibiotics were used for selections (Supplementary Table 2). Recombinants often have multiple antibiotic resistances; for instance, VZV ORFX/Kan^R^ clone will have Kan^R^, Cm^R^ (from BAC vector), and Hyg^R^ (from luciferase cassette). Screening for recombinants with more than one antibiotic is optional. However, the growth rate under such conditions could be much slower than selection under one antibiotic.

**Mini-BAC DNA preparation**

A single DY380 clone containing the putative VZV BAC was inoculated in 5ml LB supplemented with the appropriate antibiotics and cultured at 32°C overnight. BAC DNA was isolated by pelleting the bacteria, resuspending it in 1ml of resuspension buffer supplemented with RNase A (P1), lysing it in 1 ml of NaOH/SDS lysis buffer (P2), and neutralizing it in 1ml of potassium acetate neutralization buffer (P3); each step took 5 min (Nucleobond Maxiprep BAC DNA isolation kit from Clontech Laboratories, Inc., Palo Alto, CA). The cloudy solution was centrifuged at 4500g for 15min at 4°C. The supernatant was filtered through a small piece of Kimwipe (Kimberly-Clark Global Sales, Inc., Roswell, GA). The filtered solution was extracted with an equal volume of phenol/chloroform and the BAC DNA precipitated with two volumes of ethanol. After the final spin at 4500g for 30min at 4°C, the DNA pellet was resuspended in 20ul of sterile ddH_2_O.

**Maxi-BAC DNA preparation**

The large-scale BAC DNA preparations using the Nucleobond Maxiprep BAC DNA isolation kit (Clontech Laboratories Inc., Palo Alto, CA) started with 500ml of overnight cultures. Because of their large size, BAC DNAs need to be handled in a way that avoids any harsh physical shearing force, including vortexing or passing quickly through fine pipette tips. Freeze-and-thaw should also be avoided. BAC DNA solutions should always be stored at 4°C. The final DNA products were resuspended in 250ul sterile ddH2O and quantified by spectroscopy.

**HindIII (New England Biolabs, MA) digestion.** Three micrograms of BAC DNA from maxi-preparations were digested with 20U of HindIII in a 20ul reaction at 37°C overnight. HindIII digestion patterns were compared by electrophoresis on ethidium bromide-stained 0.5% agarose gels.

**Transfection**

VZV BAC DNA from Maxi-preparations was transfected into MeWo cells using the FuGene 6 transfection kit (Roche, Indianapolis, IN), according to manufacturer’s standard protocol. One-and-a-half micrograms of BAC DNA and 6ul of transfection reagent were used for a single reaction in one well of 6-well tissue culture plates. Highly concentrated (>250ug/ul) BAC DNA solutions are viscous, and BAC DNA molecules easily precipitate out of the solution when added to transfection reagent solutions. When such precipitation becomes visible, it is irreversible; predictably, the results of the transfection assays are often poor. Therefore, we pre-dilute each BAC DNA in media before gently mixing it with the transfection reagent. For each reaction, 1.5ug of BAC DNA was diluted in serum-free medium, and the volume of DNA solution was adjusted to 50ul. Using pipettor tips, we gently stirred the DNA solution into the transfection reagent. Because of GFP expression from the BAC vector, VZV plaques were usually visually discernable 3–5 days after transfection. As an option, 0.5ug of Cre expression plasmid was added during transfection to remove the BAC vector and the zeocin marker from the viral genome.
